# Social processing distorts physical distance perception

**DOI:** 10.1038/s41598-025-89935-9

**Published:** 2025-02-15

**Authors:** Georgios Michalareas, Claudia Lehr, Matthias Grabenhorst, Heiko Hecht

**Affiliations:** 1 Max Planck Institute for Empirical Aesthetics, Frankfurt am Main, Germany; 2 Psychologisches Institut, Johannes Gutenberg-Universität Mainz, Mainz, Germany; 3 Ernst Strüngmann Institute (ESI) for Neuroscience, Frankfurt, Germany; 4 CoBIC, Medical Faculty, Goethe University, Frankfurt am Main, Germany

**Keywords:** Psychology, Human behaviour

## Abstract

Psychopathy is traditionally linked to higher-level cognitive processes, but its impact on low-level cognitive functions has remained largely unexplored. This study investigates the relationship between the fundamental cognitive function of physical distance estimation and our social predisposition towards others, as captured by psychopathic traits, grouped into the three facets of Fearless Dominance, Self-Centered Impulsivity and Coldheartedness. Using an innovative experimental design, participants estimated the distance of an approaching avatar agent. We demonstrate that social processing, as reflected in the scale of Psychopathy in the general population, significantly distorts egocentric space perception. Strikingly, this distortion is absent with non-anthropomorphic agents. The study also reveals that agent emotional expressions do not affect distance estimation, when they are task-irrelevant, suggesting a disocciation between the effect of psychopathic traits on social processing and emotion processing. These results uncover a direct top-down effect of psychopathic traits on basic spatial cognition, in the presence of others. These new insights into the basic cognitive mechanisms underlying social interactions have broader implications for any field involving interpersonal spatial behaviour.

**Supplementary Information:**

The online version contains supplementary material available at 10.1038/s41598-025-89935-9.

## Main

The presence of others in our proximate space modulates our cognitive and behavioral states. This modulation entails all aspects of social processing involved in evaluating the intentions of others towards us^1^. Crucially, it is also highly dependent on our own social predisposition towards others. This predisposition is a complex construct of diverse innate and acquired personality traits^2^. These traits, which characterize our social behaviour, have been extensively studied, largely in the context of social personality disorders, with the main aim to quantify and measure the extent of these traits. One of the most prominent social personality disorders is Psychopathy. Psychopathy provides a unique lens through which to study the effects of social predispositions on cognitive functions, as its traits cluster around different dimensions that capture the degree of antisocial behavior, impaired empathy, disinhibition, and egotistical traits on an individual^3^. And most importantly these behavioral dimensions apply to the general population.

Psychopathy is a personality disorder characterized by a combination of interpersonal manipulation, affective callousness, impulsive lifestyle, and antisocial behavior, often assessed through traits such as lack of empathy, superficial charm, and disregard for social norms^4^. It is perceived by the public as the embodiment of cold and calculating behaviour, often associated with criminal or violent acts, and a propensity for lying and deceit^5^. It is a critical subject of study due to its profound implications for understanding fundamental social human behaviour and the brain mechanisms behind it. Characterized by persistent antisocial behaviour, impaired empathy, disinhibition, and egotistical traits, psychopathy affects a notable segment of the general population, with prevalence estimates of up to 4.5%^6^. Moreover, these core dimensions of psychopathy span across the general population, beyond clinical groups^7,8^. An overrepresentation of such individuals with high psychopathic traits can be found in certain professions such as politics, law enforcement, firefighting, and risky sports or military combat^9–11^. Studying these traits is necessary for understanding the general principles of interpersonal behaviour.

Research on the cognitive processes affected by psychopathy has primarily concentrated on higher-level functions, particularly emotional processing and empathy^12–14^. Neuroimaging studies with psychopathic individuals have shown significant anomalies in brain regions implicated in emotion recognition and empathy^15^. However, there has been a notable lack of research on the effect of psychopathy on basic, low-level cognitive processes, such as the estimation of spatial parameters in egocentric space. Prior studies have shown that social relations can warp distance perception^16–18^. However, the role of individual personality traits, particularly those linked to psychopathy, in modulating this effect remains underexplored. This study addresses this gap by examining whether psychopathic traits selectively influence physical distance estimation. Understanding how fundamental spatial representation is affected would provide new insights into the cognitive mechanisms underlying the disorder. This knowledge may ultimately contribute to more effective identification and treatment strategies for individuals exhibiting psychopathic traits.

Hervey Cleckley first described psychopathy in 1941 with 16 traits, emphasizing superficial charm, irresponsibility, and lack of empathy, but not criminal behaviour^19,20^. “Successful psychopaths,” who blend into society, were also included in his research. Robert D. Hare later expanded on Cleckley’s work, focusing on impulsive and manipulative traits in prisoners^21,22^. He created the Psychopathy Checklist-Revised (PCL-R), which adds criminal behaviour to Cleckley’s traits and is a risk assessment tool for offenders, though not for the general population. Following assessments, like the Psychopathic Personality Inventory-Revised^23^ (PPI-R) measure psychopathy without criminal tendencies, reflecting its broader presence in society^24,25^. The PPI-R correlates with clinical tools like the PCL-R in assessing interpersonal and affective traits and has been used in non-clinical samples, including students^26–28^.

Building on these foundational assessments, psychopathic traits have been found to be clustered around three principal dimensions, termed scales or factors of psychopathy and which are: Fearless Dominance (PPI-FD), Self-Centered Impulsivity.

(PPI-SI), and Coldheartedness(PPI-CH). Fearless Dominance includes traits like social assertiveness, stress immunity, and fearlessness, contributing to risk-taking and social dominance^23^. Self-Centered Impulsivity involves impulsivity, disinhibition, and egocentricity, reflecting a propensity to act on immediate impulses^29^. Coldheartedness denotes a lack of empathy and emotional depth, highlighting deficits in emotional processing^28,30^. These factors explain the multifaceted nature of psychopathy^25^. The large degree of independence between PPI-FD and PPI-SI has lead to the development of the influencial dual-process model of psychopathy^29^. Empathy has been the most systematically investigated aspect of psychopathy^30,31^, with the strongest evidence showing that psychopaths have impaired processing when recognizing emotional facial expressions such as fear^32^.

As psychopathy is characterized by severe deficits in social emotional behaviour, a number of studies in the past have focused on the impact of psychopathy on interpersonal spatial behaviour^27,28^. This work is typically based on the research field called ”proxemics”, which was pioneered by Hall^33^. It covers the investigation of human use of space and the effect of behaviour, communication and social interaction. Hall describes four zones of interpersonal distances that form different types of spaces: Intimate distance for embracing, touching or whispering (up to 46 cm), personal distance for interactions among good friends or family (up to 122 cm), social distance for interactions among acquaintances (up to 370 cm), and public distance used for public speaking.

Interpersonal space preferences can be modulated by personality traits affected by psychopathy. Studies indicate that individuals with higher psychopathy, and particularly its scale Coldheartedness, prefer smaller comfortable interpersonal distance^28^. Further research by Welsch and colleagues^27^ suggests that psychopathic traits influence how people adjust prefered interepersonal distance in response to facial expressions of others. Typically, people keep greater distance from angry expressions, a reaction muted in those with higher psychopathy^34,35^.

The specific features that individuals consider task-relevant when assessing interpersonal space to another agent haven’t been thoroughly examined nor the underlying brain processes. Previous work has used the subjective measure of “comfortable” or “prefered” distance, which lacks precise definition and involves complex brain processing from social cognition and emotion regulation to simple distance estimation^27,28^. Without clear parameters defining “comfortable” distance, the impact of psychopathy on this spatial judgment remains complex.

In contrast to “comfortable” distance, much more is known about the simple estimation of physical distance itself, a fundamental process in the visual system of the human brain. It is largely determined by the processing and integration of both monocular and binocular cues of the input. In the human brain, bilateral early visual areas V3A/B in the superior occipital gyrus have activations that are highly correlated with perceived distance^36^. Most importantly, these areas appear to represent perceived distance even when distance estimation is task-irrelevant. These results provide strong evidence that distance information is automatically processed in early stages of visual processing. Deficits in distance estimation can be easily behaviourally quantified. For this reason, this basic cognitive function is a good candidate for probing whether the correlates of psychopathy extend to even fundamental processes of spatial feature extraction. Some disorders can affect distance estimation. Socially anxious individuals prefer greater interpersonal distance from strangers and estimate their distance as shorter^37–40^. High acrophobia groups overestimate vertical heights to a greater degree^41^. Kim and Son^42^ studied how facial expressions affect distance estimation and found that threatening and safe expressions were judged closer than neutral ones.

Based on the above-mentioned research, the question arises whether psychopathy mainly affects high-level processing of emotional and social stimulus content or its modulatory effects extend to even more fundamental processes, such as simple distance estimation. This is still unknown and the aim of the current study is to answer this question by investigating the effect of psychopathy level on simple distance estimation.

We employed an on-screen avatar approaching the participant, who was stationary, in first-person view. The task of the participant was to stop the approaching avatar when it was at an estimated distance equal to a pre-cued target distance. The factor distance was parameterized in 6 different levels between 0.5 and 3.6 m. The second parameterized factor was the facial expression of the approaching avatar, which could be happy, angry or neutral. The psychopathy level of each participant was assessed by the PPI-R questionnaire. The primary aim was to investigate whether and how psychopathy level modulates the deviation of the estimated distance from the true target distance. Here distance was the task-relevant feature and thus if psychopathy had effect in such low-level features, it was expected to be manifested in the distance estimation error. Regarding the effect of the facial expression of the avatar on simple distance estimation, the expectations were mixed. On one hand facial expression was not task-relevant here and could have received little attention by the brain to have an effect at all. On the other hand, according to Welsch and colleagues^27^, in the context of estimation of “comfortable” interpersonal distance, facial expression was considered peripheral information and still should have an effect, being highest in low psychopathy individuals. If this was indeed the case, one could expect facial expression to have a similar effect also in the simple estimation of interpersonal distance. Finally, an identical second experiment was performed, where the avatar was replaced by a non-human-resembling object in order to study whether the fundamental social factor of human resemblance produces a different modulatory effect of psychopathy on distance estimation.

Overall, this work has three novel contributions: (1) the study of how social predispositions affect the basic cognitive function of distance estimation, independent of complex concepts like “comfort” or “preference”; (2) the investigation of whether this effect on distance estimation is modulated by emotional content (facial expressions) when it is not task-relevant; and (3) the examination of whether this effect depends on the social factor of human resemblance of the approaching object. These contributions significantly enhance our understanding of how social processing factors influence fundamental cognitive functions, extending beyond previously assumed high-level processes to more basic low-level computations in the human brain.

## Results

### Distribution of psychopathy score

**Fig. 1:**
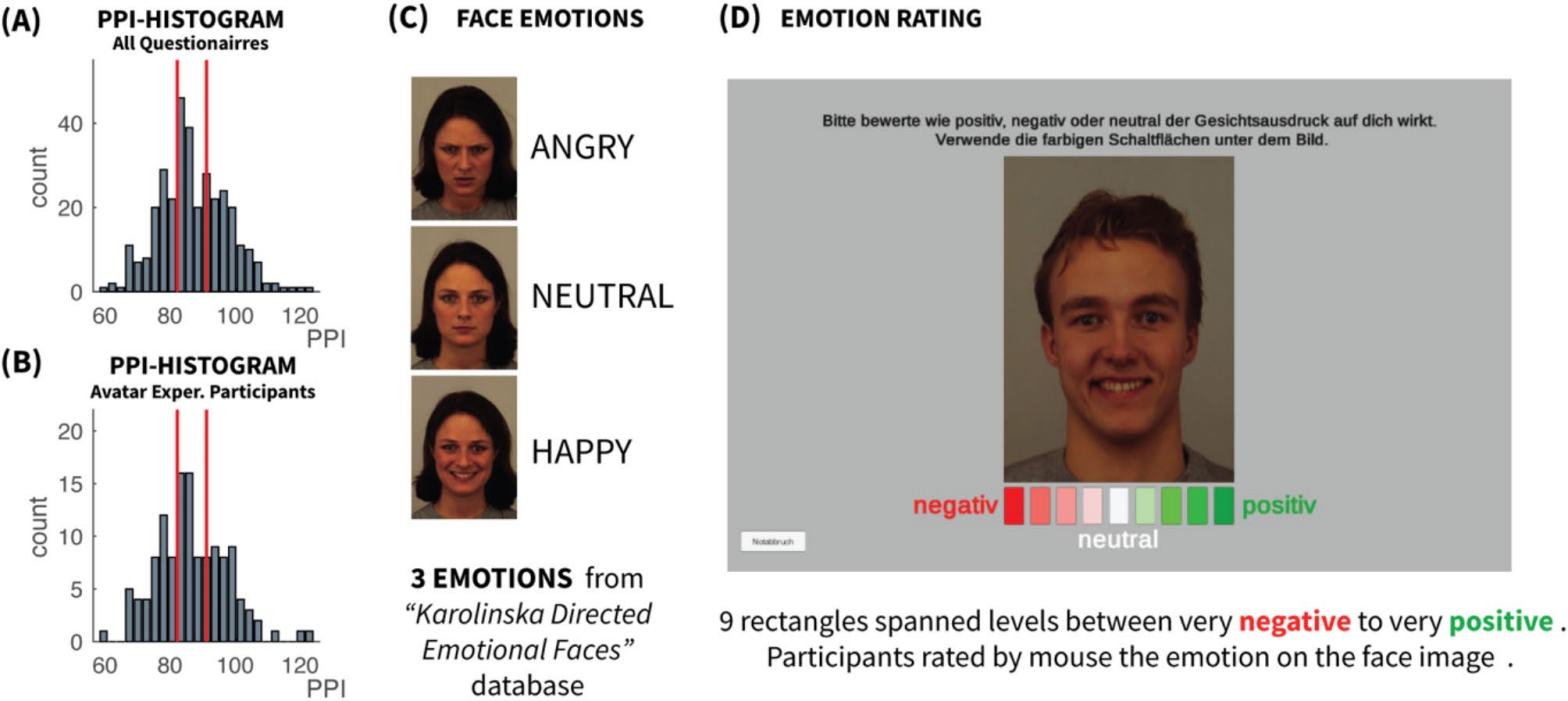
Rating of face expressions. (**A**) Histogram of PPI for all participants from the initial Questionnaire phase. (**B**) Histogram of PPI for the subset of participants who performed the Avatar experiment. In both (A) and (B) the two vertical red lines represent the 33.33% and 66.66% terciles that were used to define three psychopathy (PPI) groups (low, medium, high). (**C**) Face images were selected with three different emotions from the “Karolinska Directed Emotional Faces” database (see Methods). (**D**) Participants had to rate the face expression on an image by selecting a mark on a 9-point scale ranging from negative (red) to positive (green). All face images in the figure are from actors and actresses of the “Karolinska Directed Emotional Faces” database (see Methods).

The first, preliminary step of the investigation was the study of the distribution of psychopathic traits in the population. A cohort of 336 recruited participants were asked to complete the PPI-R self-assessment questionnaire, which is comprised of eight subscales loaded in three different factors namely Fearless Dominance, Self-Centered Impulsivity and Coldheartedness (see Methods). The sum of the scores of these factors is used as an overall psychopathy score, which henceforth will be referred to as PPI index. Higher values of PPI represent higher extend of psychopathic traits and vice versa. PPI had a median of 86 (SD = 10.7) with a slightly positive skewness (0.27) and terciles of 82 and 91.5. This distribution, depicted in Fig. 1

Figure 1A served as reference for the subsequent parts of the study.

### Avatar - experiment

The questionnaire participants were reinvited to perform a distance estimation experiment online. A total of 128 participants accepted the invitation. The PPI distribution closely mirrored that of the broader group (see Fig. 1B), with a median of 85 (SD = 11.2), positive skewness (skewness = 0.44) and terciles at 82 and 90.8. According to the terciles of the initial broader questionnaire cohort, the participants were divided into three psychopathy groups (low, middle, high). The datasets from 18 participants were excluded during data quality assessment, due to failing to comply with some of the acceptable quality criteria (See Methods). The datasets of the remaining 110 participants were further analyzed.

The experiment tasked participants with stopping an approaching avatar with one of three emotional expressions (happy, angry, neutral), sourced from the “Karolinska Directed Emotional Faces” database (example shown in Fig. 1B), at a pre-cued distance. In the initial stage of the experiment participants were asked to rate the emotional valence of 24 such facial expressions in order to validate the conveyed emotions. The rating interface is depicted in Fig. 1C. These ratings were analyzed using ANOVA with psychopathy level (low, middle, high) as a factor to explore its effect on emotion perception. There was a significant main effect of emotion (Greenhouse–Geisser F(1.5, 187.0) = 2020.87, *p* < 0.001, η² = 0.94) without any notable influence from psychopathy level (F(2, 125) = 0.82, *p* = 0.442), indicating consistent emotion recognition across psychopathy groups.

**Fig. 2:**
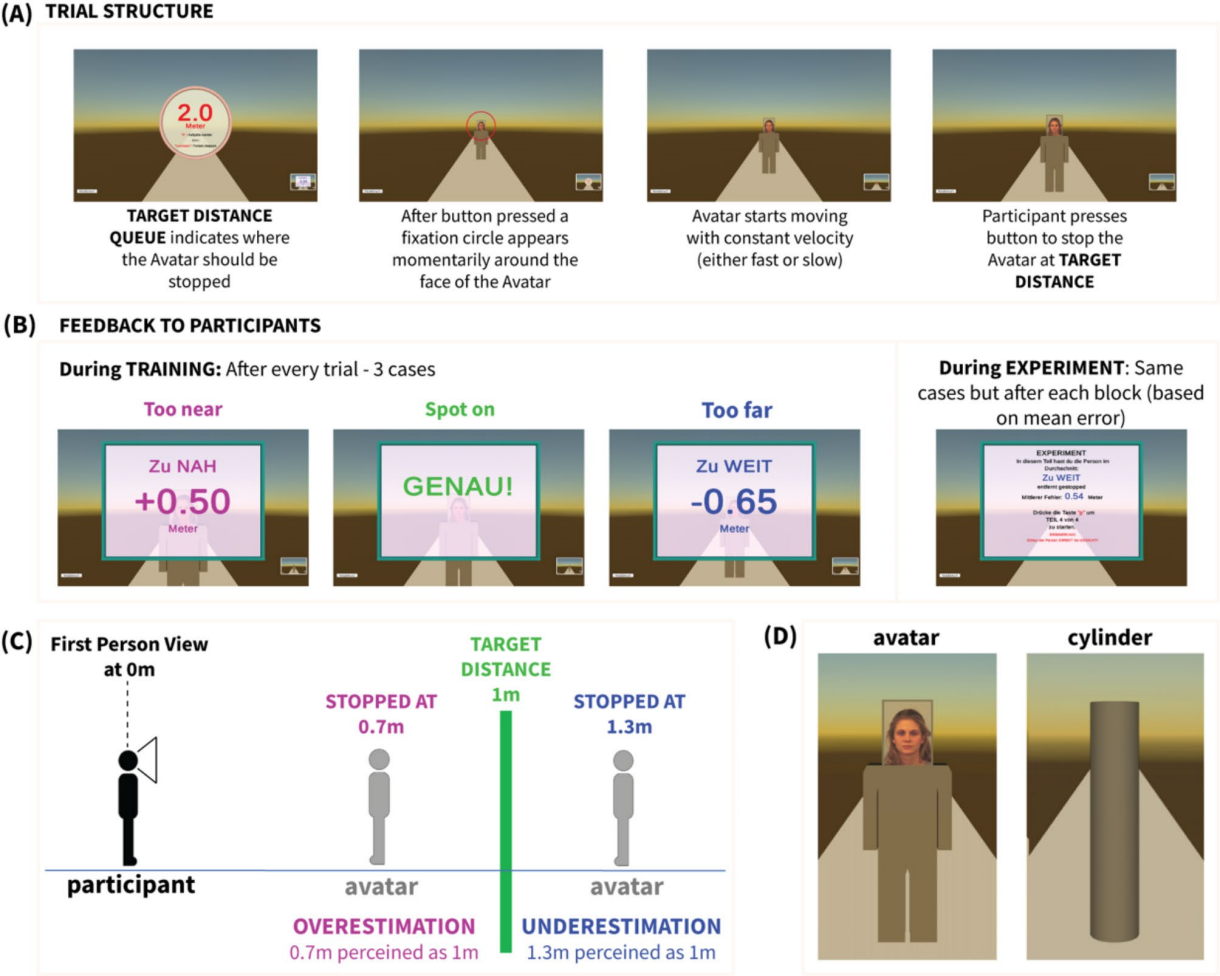
Distance estimation task. (**A**) Single trial structure. At the beginning of each trial an instruction screen displayed the target distance where the avatar should be stopped. The task started with a participant button press. The avatar was presented in its initial position and a fixation marker (red ring with small sphere in center) was momentarily used to focus the participant’s attention on the face of the avatar. The avatar started moving towards the participant, who had to stop it by pressing the space bar at the previously displayed target distance. With the participant’s button press, the avatar stopped instantly. The participant had to press a button to proceed to the next trial. The same trial structure was used both in training and in experiment blocks. (**B**) During training, feedback was provided after each trial to the participant about the error in their distance estimation w.r.t to the target. There were three cases of feedback. Too near, Too far and Spot on (“zu nah”, “zu weit” and “spot on” in German). Similar feedback was provided also during the experiment, but only at the end of each block, based on the average error across all the trials of the block. (**C**) Intuitive description of distance overestimation and underestimation when the participants stopped the avatar too near or too far respectively. (**D**) Object experiment. The avatar was replaced by a cylinder with dimensions coinciding with the head dimensions of the avatar. Here also the avatar is displayed for comparison.

The main part of the experiment consisted of a first training session and subsequent 4 blocks of the actual task (see Methods). The trial structure was similar for both training and actual experiment parts (Fig. 2A). At the beginning of each trial a *target* interpersonal distance, $\:{\theta\:}_{{\rm\:T}}$, was presented briefly to the participant. Then the avatar, with one of the three facial expressions, started approaching the participant from a distance of 5.5 m, who had to stop it at her/his *estimated target* distance $\:{\theta\:}_{E}$. The avatar moved with one of two possible speeds (slow, fast), in order to focus the attention of the participants to distance rather than elapsed-time estimation with a single fixed speed. The training block aimed to familiarize the participants with the dimensions of the virtual environment employed, with feedback-guided learning. Feedback, shown in Fig. 2B, was provided at the end of each trial only in the training session. In the main experiment, feedback was given only at the end of each block, about the average performance of the participant in this block. The primary focus was on analyzing the distance estimation error $\:\varDelta\:\theta\:={\theta\:}_{E}-{\theta\:}_{T}$, with positive errors when avatar stopped further (underestimation), and negative errors when stopped nearer (overestimation) than the target distance. For example, stopping the avatar at $\theta _{{\rm E}} = 1.3m$, while believing that it is at a target distance of $\theta _{T} = 1m$ was considered a distance underestimation with a positive error $\Delta \theta = 0.3m$. The experimental design and its foundational principles are elaborated in Fig. 2C. The avatar employed in this experiment is shown in Fig. 2D.

### Avatar – mixed effect ANOVA

To investigate the influence of psychopathy and avatar facial emotion on distance estimation, a mixed-design ANOVA was performed, with distance error $\:\varDelta\:\theta\:$ as the dependent variable. Facial expression (neutral, happy, angry) was a within-subject factor, while psychopathy level (low, mid, high) acted as between-subject factor. Avatar speed was used as a second within-subject factor due to its direct modulation of the distance error, as for the same reaction time of the participant upon the decision to stop the avatar, the avatar would travel longer distance with fast speed.

Across participants, the mean distance error was 12.51 cm (SEM = 1.55 cm) and 95% confidence intervals at [9.41 15.6] cm. This significant positive error offset was confirmed by a Wilcoxon signed-rank test (Z = 6.47, *p* < 0.0001) which means that the Avatar was stopped in the majority of cases further than the target distance (underestimation).

**Fig. 3:**
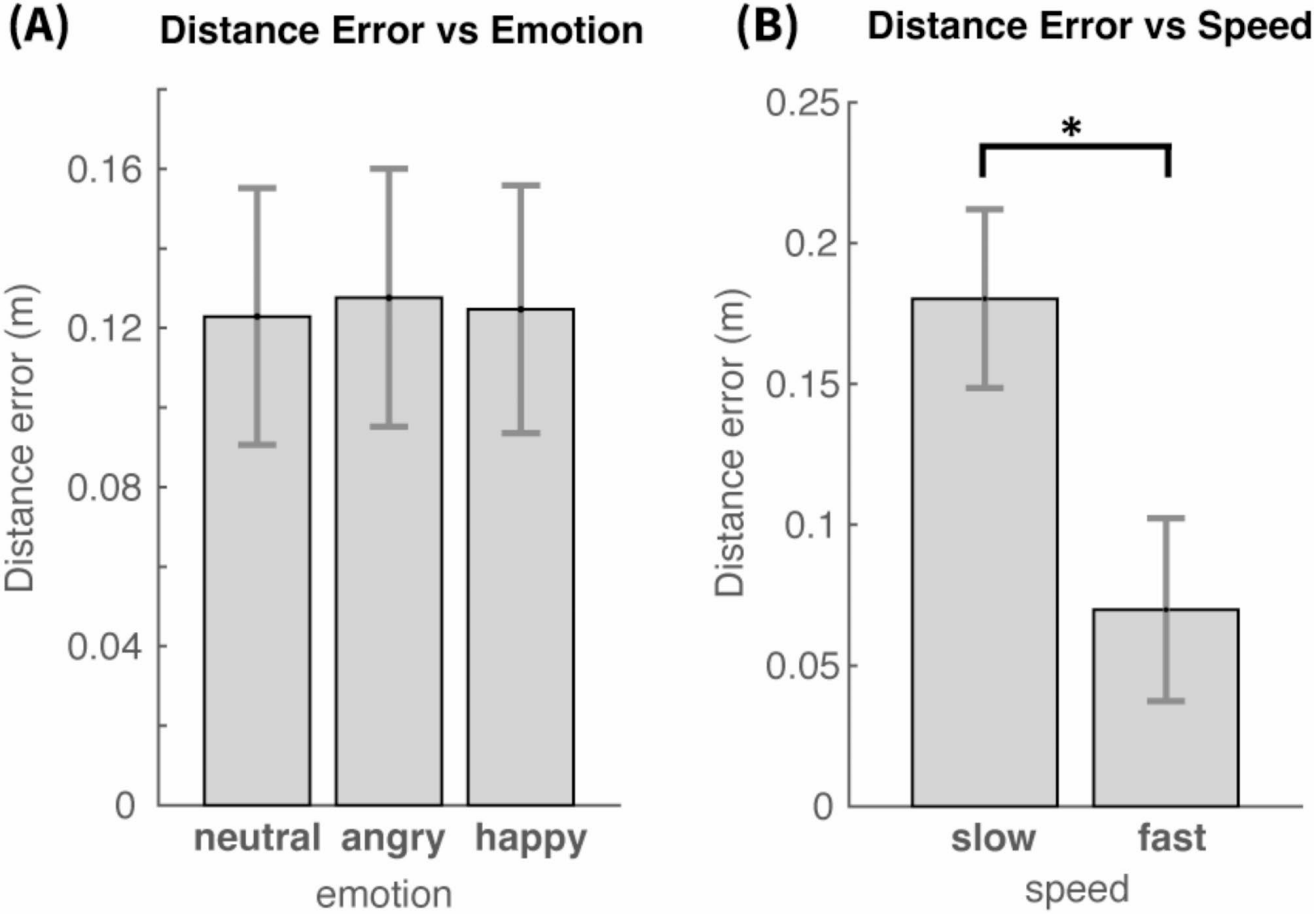
Distance error vs. emotion and speed. (**A**) Distance error vs. emotion. Mean and 95% confidence intervals of distance estimation error across participants for each facial emotion type. No significant effect of emotion was found. (**B**) Distance error vs. speed. Mean and 95% confidence intervals across participants for slow and fast speed. There was a significant effect of speed. The mean distance error was much larger for slow speed.

Facial Emotion was not found by the ANOVA to have any significant effect on distance error (F(2, 214) = 0.16, *p* = 0.85). The mean distance error with corresponding SEM for each facial emotion is shown in Fig. 3A. This finding demonstrated that emotional cues from the avatars did not influence participants’ spatial judgments.

The factor Speed had significant effect on distance error (F(1, 107) = 167.73, *p* < 0.0001, η² = 0.06). With slow speed, distance error was 11 cm larger (*p* < 0.001) than with fast speed, as shown in Fig. 3B (Slow: $\:\overline{\varDelta\:\theta\:}$= 0.180, SEM = 0.016, Fast: $\:\overline{\varDelta\:\theta\:}$=0.070, SEM = 0.016 m). This means that the avatar was stopped closer to the participant when the avatar was moving faster, as expected.

**Fig. 4:**
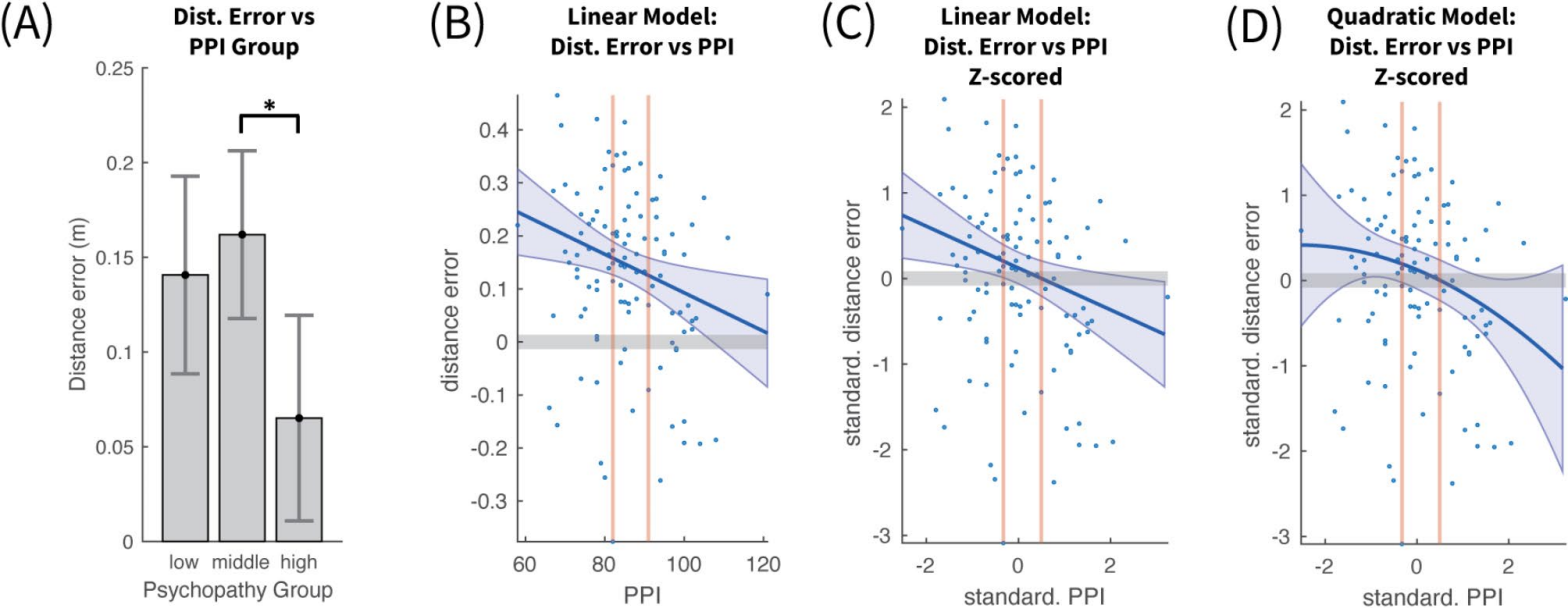
Distance vs. psychopathy personality inventory (PPI). (**A**) Mean and 95% confidence intervals of distance estimation error across participants for each PPI group. Statistical significance was reached only for the difference between middle and high PPI groups. (**B**) Linear fit between PPI and Mean Distance Error of each participant. The shaded area around the fitted line represents the 95% confidence area of the fit. The red vertical lines represent the terciles of distance estimation error. Notice that the distance error is predominantly positive across the entire range of PPI. (**C**) shows the same data as in (**B**), but with both PPI and Mean Distance Error standardized using z-scores. (**D**) Fit by a quadratic polynomial between the standardized PPI and Mean Distance Error of each participant.

The ANOVA confirmed a main effect of Psychopathy level F(2, 107) = 4.18, *p* < 0.05, η² =0.038. The mean distance error for each group is shown in Fig. 4A. The low and middle psychopathy groups exhibited similar estimation errors (14.7 cm and 16.1 cm respectively), whereas high psychopathy individuals demonstrated significantly lower errors (6 cm). Post-hoc analysis highlighted a significant difference in estimation errors between the middle and high psychopathy groups, with a difference of 9.5 cm (*p* < 0.05). The difference between the low and high psychopathy group was comparable at 8 cm but it did not reach statistical significance (*p* = 0.065), with a Bonferroni multiple comparison correction. The main underlying cause for this could be some form of inverted asymmetric U-shape relation between the distance error and PPI index, so that the peak error occurs in the middle group and it drops in either extremum. Such a pattern could be revealed by using parametric modelling of the distance estimation error based on the PPI values themselves and not their discretization into three groups.

### Avatar – polynomial models of distance estimation error vs. PPI

To analyze the relationship between PPI and distance error $\:\varDelta\:\theta\:$, first a linear model was employed of the form $\:\varDelta\:\theta\:=\beta\:\cdot\:PPI+\gamma\:$. The regression revealed a significant inverse relation between PPI and $\:\varDelta\:\theta\:$ (R^2^ = 0.074, F(1, 108) = 8.62, *p* = 0.004), with negative slope (slope β = −0.00363, *p* = 0.01; intercept γ = 0.455, *p* = 0.0002). This model (Fig. 4B), confirmed the positive distance error offset across all PPI values. A linear model fitted on the z-scores of $\:\varDelta\:\theta\:$ and $\:PPI$, shown in Fig. 4C, provided a more interpretable slope coefficient (slope β = -0.244, *p* = 0.01; intercept γ = 0.123, *p* = 0.188), verifying the strong anticorrelation.

In order to investigate whether some form of inverted U-shape model would capture better the relationship between $\:\varDelta\:\theta\:$ and $\:PPI$, a quadratic model ( $\:\varDelta\:\theta\:=a\cdot\:{PPI}^{2}+\beta\:\cdot\:PPI+\gamma\:)$ was fitted on their z-scores. The model (Fig. 4D) was statistically significant (R^2^ = 0.08, F(2, 107) = 4.65, *p* = 0.012), with a similar significant negative slope as the linear model (linear coef. β = -0.225, *p* = 0.02). The quadratic coefficient (α = −0.044, *p* = 0.50) had a small negative value, giving to the model a subtle downward concave shape, but this value was not found to be significantly different than zero. The slightly higher R^2^ of the quadratic model can be interpreted as explaining a slightly larger amount of variance compared to the linear model. In conclusion there was no evidence of significant flattening or inversion of the linear negative slope towards the low PPI range.

### Avatar – self-centered impulsivity (PPI-SI)

In order to discern which PPI subscales most influence the negative gradient of distance error with PPI, the repeated measures ANOVA was conducted again but with the PPI index replaced by its three subscales, Coldheartedness (PPI-CH), Fearless Dominance (PPI-FD), and Self-Centered Impulsivity (PPI-SI) as between-subject factors. Speed and Emotion remained the within-subject factors. For the ANOVA, Fearless Dominance and Self-Centered Impulsivity were each discretized into 3 groups (low, middle, high) according to their respective terciles. The estimated terciles were [34,39] for PPI-FD and [38,43] for PPI-SI. Coldheartedness, due to its narrow integer range of [5.00 19.00], was discretized into two groups (low, high) based on its median value of 9. The above mentioned terciles and median values were estimated from the entire initial questionnaire cohort of 336 participants.

**Fig. 5:**
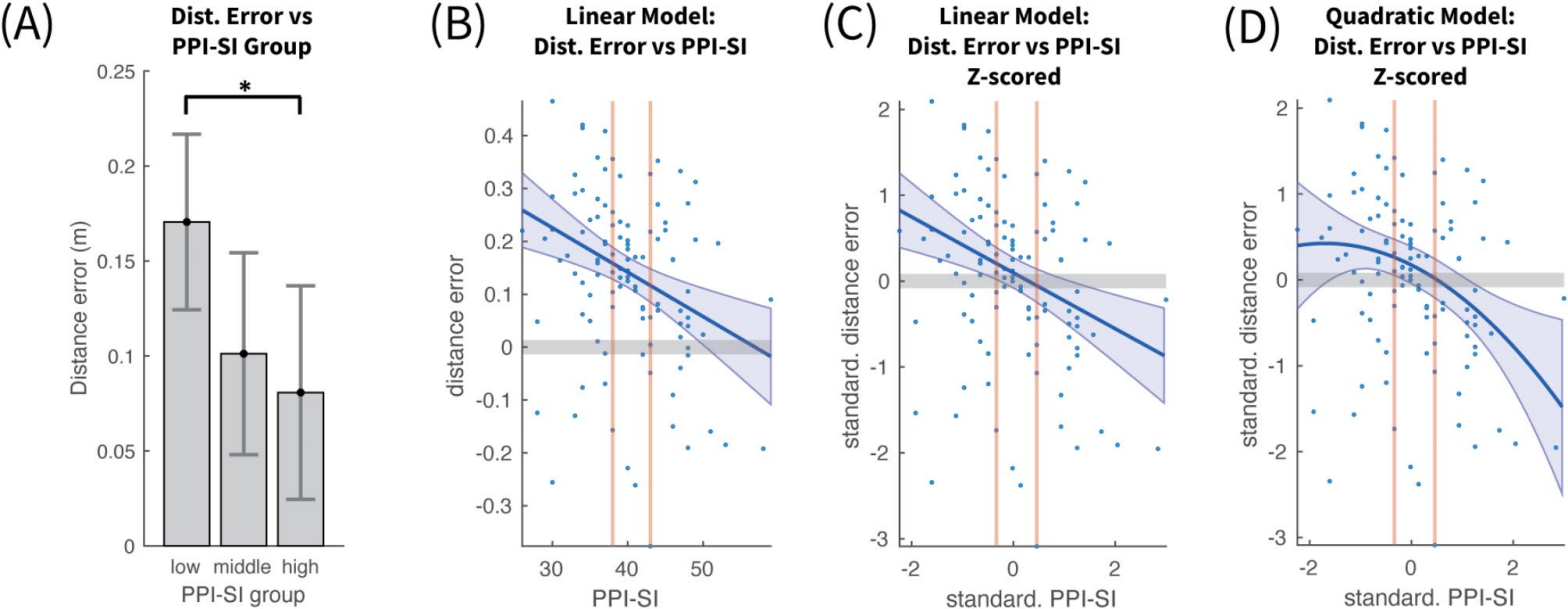
Distance vs. self-centered impulsivity subscale (PPI-SI). (**A**) Mean and 95% confidence intervals of distance estimation error across participants for each PPI-SI group. Statistical significance reached only for the difference between low and high PPI-SI groups. (**B**) Linear fit between PPI-SI and Mean Distance Error of each participant. The shaded area around the fitted line represents the 95% confidence area of the fit. The red vertical lines represent the terciles of distance estimation error. (**C**) Same as in (**B**) but with the PPI-SI and Mean Distance Error standardized through z-scores. (**D**) Fit by a quadratic polynomial between the standardized PPI-SI and Mean Distance Error of each participant.

From the three subscales of PPI only Self-Centered Impulsivity(PPI-SI) showed a significant effect on distance errors (F(2, 107) = 3.59, *p* = 0.032, η² = 0.037), with no significant impact from Coldheartedness (F(1, 108) = 0.08, *p* = 0.772) or Fearless Dominance (F(2, 107) = 0.17, *p* = 0.837). Post-hoc analysis highlighted a significant difference in distance errors only between the low and high PPI-SI groups (10.28 cm, *p* < 0.05), with all groups having positive distance error as shown in Fig. 5A.

Similar to the PPI case, parametric models were employed to fit the relation between distance error $\:\varDelta\:\theta\:$ and PPI-SI itself, without any discretization into groups. First, linear regression on $\:\varDelta\:\theta\:$ and PPI-SI (Fig. 5B), demonstrated a statistically significant inverse relationship (R² = 0.118, F(1, 108) = 14.47, *p* < 0.001), with a slope of β = -0.0084 (*p* = 0.0004) and intercept γ = 0.477 (*p* < 0.0001). Α linear model was fitted to the z-scores of $\:\varDelta\:\theta\:$ and PPI-SI (Fig. 5C) to provide a more interpretable slope coefficient (slope β = -0.325, *p* = 0.0004; intercept γ = 0.09, *p* = 0.279) which made clear the strong anticorrelation. This significant negative slope, comparable to that of PPI index (β = −0.225, *p* = 0.02) seen earlier, indicates that the PPI-SI is the main driving factor behind PPI, while the other subscales rather introduce noise to this relation.

Quadratic polynomial modeling of $\:\varDelta\:\theta\:$ and PPI-SI z-scores, aimed at assessing deviations from the linear model, yielded a significant regression (R² = 0.16, F(2, 107) = 10.2, *p* < 0.0001) but with a non-significant quadratic coefficient (α = -0.088, *p* = 0.167), suggesting a primarily linear influence of PPI-SI on distance estimation error (Fig. 5D).

### Cylinder - experiment

The Avatar experiment raised two main questions regarding distance estimation errors. First, despite the absence of a significant effect from the avatar’s emotional expression on these errors, it remained unclear what stimulus attribute interacted with psychopathic traits in order to modulate these errors (negative slope). In this context, the only relevant attribute was the human-like appearance of the stimulus. Second, the consistently positive distance error in the entire PPI range, combined with its negative slope across it indicates that individuals with higher psychopathy may estimate distances more accurately (smaller positive error). However, such a consistent positive error could also be due a corresponding estimation error offset due to the characteristics of the 2D virtual environment used.

**Fig. 6:**
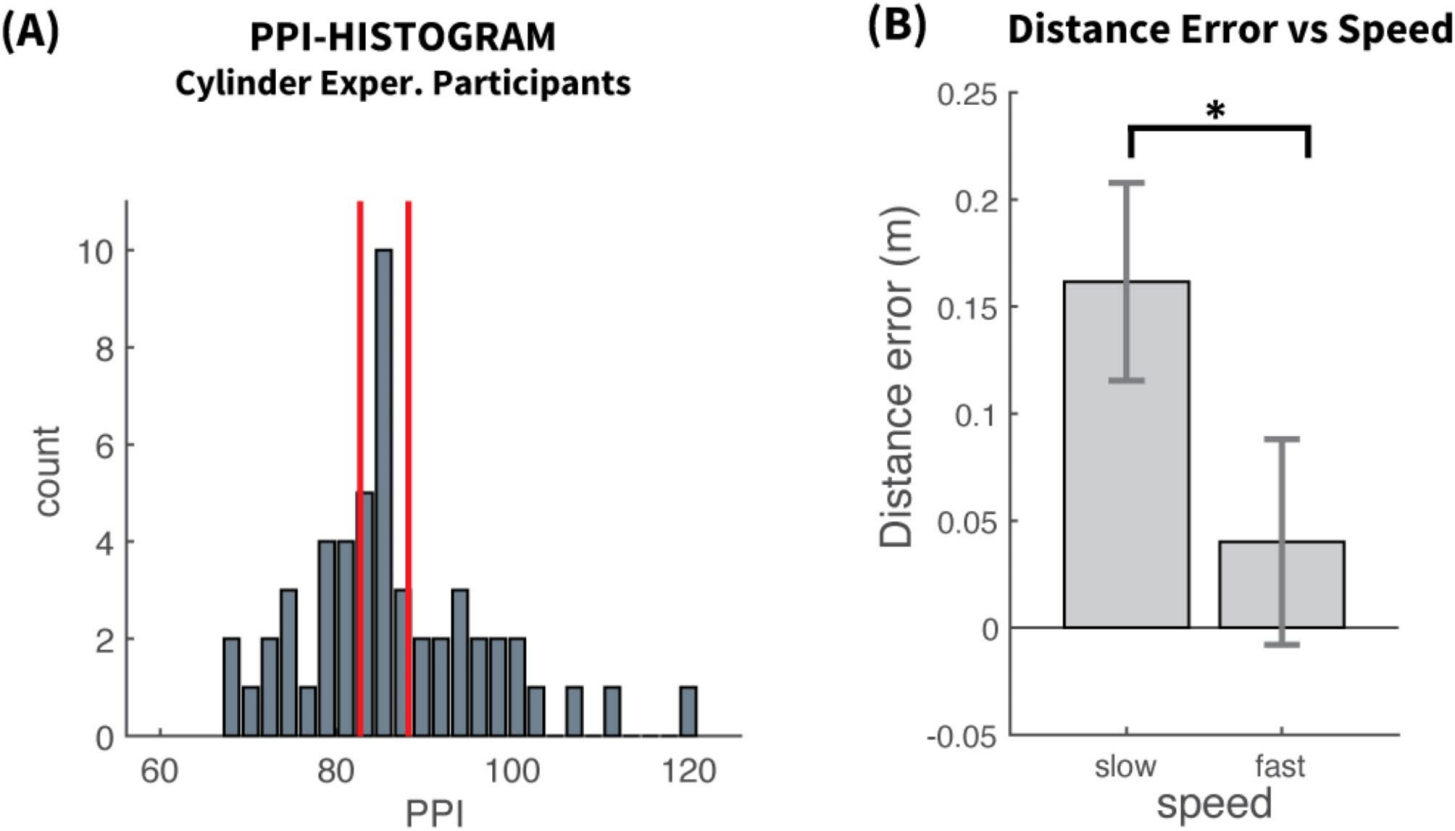
Cylinder experiment. (**A**) Histogram of PPI for the subset of participants who performed the Cylinder experiment. The red lines represent the terciles. The distribution is in resemblance with those from the Questionnaire and the Avatar experiment in Fig. 1. (**B**) Distance Error vs. Speed. Mean and 95% confidence intervals across participants for slow and fast speed. The distance error was significantly much larger for slow speed, as in the Avatar case.

In order to explore these issues further, a follow-up experiment replaced the avatar with a non-human-resembling cylinder (Fig. 1D), eliminating the influence of human resemblance. This study aimed to determine if removing human features would affect the modulation of distance error by psychopathy. All participants from the original study were re-invited, 53 of which participated, with data from one excluded in quality assessment. This left 52 participants whose PPI distribution resembled those in the initial experiment (Fig. 6A) with median 85 (SD = 10.8), skewness = 0.78 and terciles [82.67, 88.17].

The trial structure and analysis steps were identical to the avatar experiment, with the only difference being the absence of facial emotional expressions, both as stimulus features and as potential factor of distance error modulation.

### Cylinder – mixed effect ANOVA

A mANOVA evaluated the impact of psychopathy group on distance error $\:\varDelta\:\theta\:$, with psychopathy group (low, middle, high) and speed (slow, fast) as between- and within-subject factors.

Speed significantly affected $\:\varDelta\:\theta\:$ (F(1, 50) = 64.51, *p* < 0.0001, η² = 0.088) in a way identical to the Avatar case. With slow speed, $\:\varDelta\:\theta\:$ was 12.4 cm larger (*p* < 0.0001) than with fast speed, as shown in Fig. 6B (Slow: $\:\stackrel{-}{\varDelta\:\theta\:}$= 0.162, SEM = 0.023, FAST: $\:\stackrel{-}{\varDelta\:\theta\:}$= 0.040, SEM = 0.024 m).

**Fig. 7:**
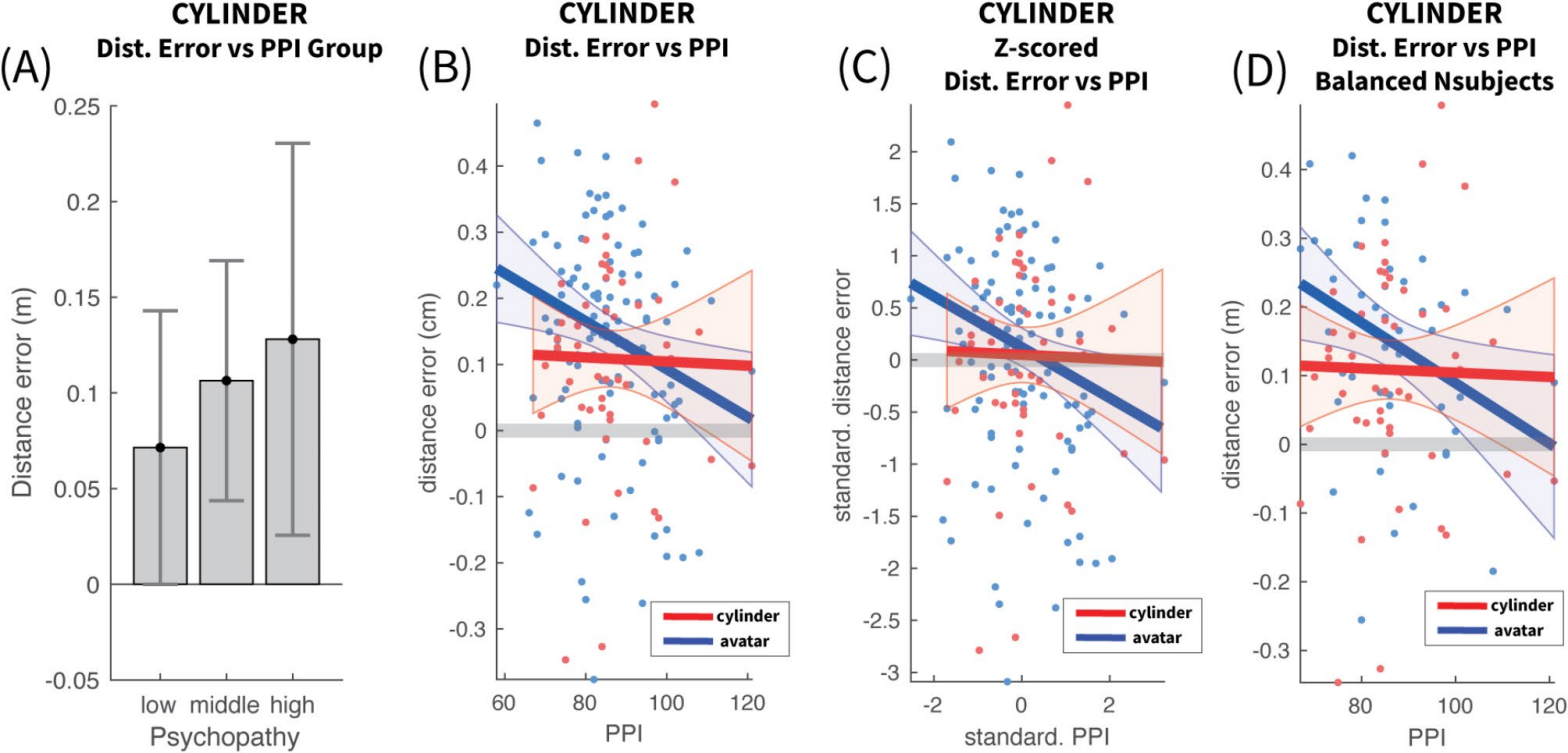
Distance vs. PPI for cylinder stimulus. (**A**) Mean and 95% confidence intervals across participants for each PPI group. No statistical significance was reached between any of the PPI groups. (**B**) Linear models of the raw distance estimation error and PPI values for the Cylinder (red) and Avatar (blue) cases. The shaded area around the fitted line represents the 95% confidence area of the fit. The blue line is the linear fit for the Avatar case, already presented in Fig. 4B, and has been replotted here for direct comparison with the Cylinder case. Clearly the slope for the Cylinder case is much flatter and not significantly different than 0, in contrast to the Avatar which has a significant negative slope. The two models have a similar distance error offset, with the intersection towards the upper end of PPI (intersection at PPI = 96.3). (**C**) Linear fit between standardized (Z-scored) PPI and Mean Distance Error. The standardization has removed the offset in both models. In such case the intersection is moved more towards the mean (intersection at PPI = + 0.32z = 89). (**D**) Same as in (**B**) but with the Avatar linear model fitted on data only from the participants who also participated in the Cylinder experiment. No significant changes are seen.

In contrast to the Avatar case, psychopathy group had no effect on distance error (F(2, 50) = 0.488, *p* = 0.617, η² = 0.012). Figure 7A presents the mean error for each psychopathy group (Low: [$\:\stackrel{-}{\varDelta\:\theta\:}$=0.072, SEM = 0.036], Middle: [$\:\stackrel{-}{\varDelta\:\theta\:}$=0.106, SEM=0.032], High: [ $\:\stackrel{-}{\varDelta\:\theta\:}$=0.128, SEM=0.052]). It is comparable for all groups, demonstrating that the removal of human-resemblance has abolished the effect of psychopathy on distance estimation error.

Furthermore, similar to the Avatar case, $\:\varDelta\:\theta\:$ was consistently positive across the PPI range with a mean of 10.08 cm (SEM = 2.2 cm), confirmed by a Wilcoxon test (Z = 4.13, *p* < 0.0001). The persistence of this positive offset for a non-human-resembling object, combined with the absence of any modulation by psychopathy group, indicated a positive bias due to the VR environment used. In this case this offset, termed $\:{\varDelta\:\theta\:}_{B}$, corresponds to an actual zero distance estimation error, when the target distance was accurately estimated by the participant. Any smaller $\:\varDelta\:\theta\:$ should thus be considered as an actual negative error and larger $\:\varDelta\:\theta\:$ as positive.

### Cylinder – polynomial models of distance estimation error vs. PPI

Linear regression evaluated the relationship between $\:\varDelta\:\theta\:$ and PPI index, without discretization into groups. The regression was not statistically significant (R² = 0.003, F(1, 50) = 0.159, *p* = 0.692). The slope coefficient had a very small value, not significantly different than zero (β = −0.0003, *p* = 0.877, intercept γ = 0.135, *p* = 0.4377). This nearly-flat curve is illustrated in Fig. 7B in red, accompanied for convenience by the Avatar’s curve in blue, with the significant negative slope. These curves intersect at PPI value of 96.3. The distance error at this PPI level should be considered as the method bias offset $\:{\varDelta\:\theta\:}_{B}$. Under this assumption, in the Avatar case for higher psychopathy the error becomes negative (stopped nearer than target) while for lower psychopathy the error is positive (stopped further than target).

A linear model on the z-scores of $\:\varDelta\:\theta\:$ and PPI for the cylinder case gave a more interpretable value of the slope coefficient, which confirmed its minuteness (β = −0.02, *p* = 0.8765; γ = 0.048, *p* = 0.715). This model is presented in Fig. 7C, accompanied by corresponding model for the Avatar case. Their intersection has moved to the left at PPI = + 0.3*z = 89.7, moving lower than the second tercile.

Due to the higher number of participants in the Avatar case, its linear model was fitted again with the data only from the smaller cohort that also participated in the cylinder experiment. The results were identical with the original model (β = -0.0044, *p* = 0.02; γ = 0.53, *p* = 0.0017), shown in Fig. 7D. Additionally, a linear model was fitted to the corresponding z-scores in order to extract a more interpretable slope coefficient, which was found to be β = -0.326 (*p* = 0.02).These results verified, that the abolition of the negative slope in the cylinder case was not due to the smaller sample.

Finally in order to directly compare the slope for the Avatar and the Cylinder cases, we fitted a linear model to the paired differences of Avatar-Cylinder distance errors for the smaller cohort that performed both experiments. The results showed a comparable slope to that of the Avatar, which was statistically significant (β = -0.326, *p* = 0.036). This test confirmed that significant negative slope in the Avatar case and the near flat slope in the Cylinder case.

### Cylinder – self-centered impulsivity

**Fig. 8:**
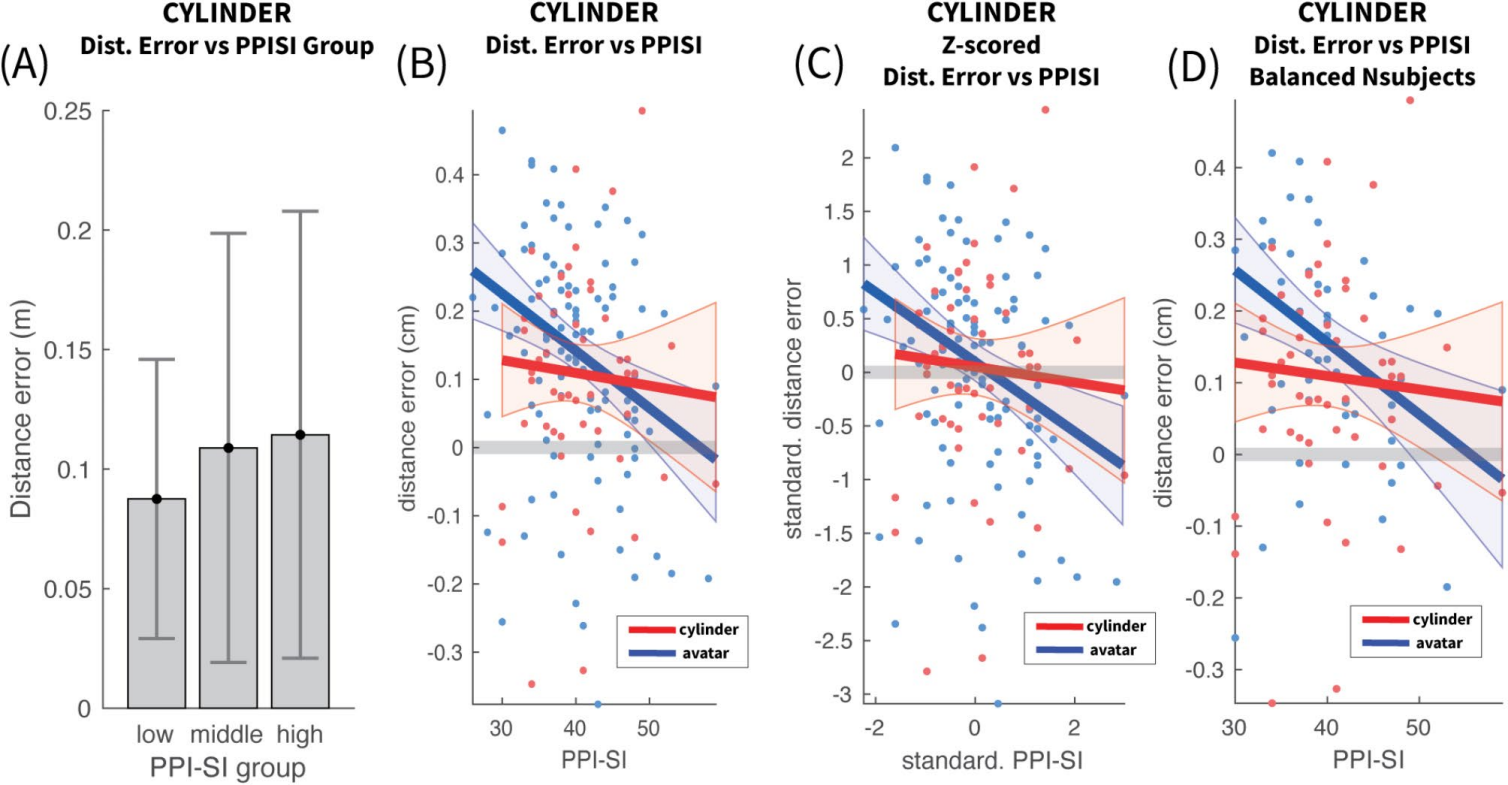
Distance vs. PPI-SI for cylinder stimulus. (**A**) Mean and 95% confidence intervals across participants for each PPI-SI group. No statistical significance was reached between any of the PPI-SI groups. (**B**) Linear models of the raw distance estimation error and PPI-SI values for the cylinder (red) and Avatar (blue) cases. The shaded area around the fitted line represents the 95% confidence area of the fit. The blue line is the linear fit for the Avatar case, already presented in Fig. 5B, and has been replotted here for direct comparison with the Cylinder case. The slope for the Cylinder case is much smaller and not significantly different than 0, in contrast to the Avatar which has a significant negative slope. The two models have a similar distance error offset, with the intersection towards the upper end of PPI-SI (intersection at PPI-SI = 44.9). (**C**) Linear fit between standardized (Z-scored) PPI-SI and Mean Distance Error. The standardization has removed the offset in both models. In such case the intersection is moved more towards the mean (intersection at PPI-SI = + 0.17z = 41.2). (**D**) Same as in (**B**) but with the Avatar linear model fitted on data only from the participants who also participated in the Cylinder experiment. No significant changes are seen.

In contrast to the Avatar case, a repeated measures ANOVA indicated no significant PPI-SI group effect on distance error in the Cylinder case (F(2, 50) = 0.240, *p* = 0.788, η² = 0.006). Distance errors were comparable across the three PPI-SI groups, with low, middle, and high psychopathy groups exhibiting mean errors of 7.96, 11.5 and 10.8 cm, respectively, shown in Fig. 8A.

Linear regression analysis further confirmed that PPI-SI did not predict distance error (R² = 0.0088, F(1, 50) = 0.444, *p* = 0.508), with a very small slope coefficient ( slope β = -0.0019, intercept γ = 0.184), as seen in Fig. 8B A linear model fit to the z-scores of$\:\:\varDelta\:\theta\:$ and PPI-SI, shown in Fig. 8C, made the slope coefficient more interpretable and confirmed the very low correlation (slope β = -0.07, *p* = 0.594). The Avatar linear model was refitted with only data from the reduced cohort of the cylinder experiment in order to verify that the abolition of the negative slope was not to the reduced sample. The Avatar model, as shown in Fig. 8D, remained identical to the one from the cohort case.

Finally, a linear model was fitted to the paired differences of Avatar-Cylinder distance errors, in order to directly compare the slope for the Avatar and the Cylinder cases. The results showed a comparable slope to that of the Avatar, which approached significance but did not cross the threshold (β = -0.303, *p* = 0.087). Given that PPI-SI is a subscale of PPI, for which the slope of the difference was significant, we attribute this near-significance into the statistical power required to quantify more accurately the small slope in the Cylinder case, rather in identifying the large slope in the Avatar case.

### Interaction of psychopathy with distance

As a final step we tested whether distance itself interacts with Psychopathy, in order to understand if the main effect comes from a specific psychopathy group and/or a specific distance range. For this purpose, the same ANOVA analysis was repeated for the Avatar and the Cylinder cases by adding an additional within-subject factor, Distance location. The results showed that there was no significant interaction between PPI and Distance. The results are presented in Supplementary Information in the section “Effect of Distance on distance estimation error”, where Figure S2 shows also the distance estimation error per location.

## Discussion

Psychopathic traits play a crucial role in modulating social processing in the human brain. While research has traditionally focused on higher-level processes such as emotional and social cognition, this study extends our understanding of how psychopathic traits influence basic spatial perception. We have demostrated a selective association between these traits and distance estimation in socially relevant contexts, highlighting the unique interplay between psychopathy and fundamental spatial cognition.

When approached by an Avatar, psychopathic traits had a significant effect on distance estimation, best captured by a linear model with a negative gradient across PPI values. In contrast the emotional expression of the Avatar had no effect on distance estimation. When the Avatar was replaced by a non-anthropomorhic object, a cylinder, the psychopathy effect vanished, showing that human resemblance is key in distance perception. When it was further explored which psychopathic traits affect distance estimation, it was found that only Self-Centered Impulsivity had a significant effect, solely explaining the negative gradient of distance error with respect to PPI.

The absence of any modulation of distance estimation by the Avatar’s emotional expression contrasts with previous findings from Welsch and colleagues^43^. In that study participants had to stop at a “comfortable” distance to the Avatar, making emotion task-relevant. Here, with a simple distance estimation task, emotion was irrelevant to the task. The Coldheartedness factor, which influences interpersonal distance when emotion is relevant^44,45^, had also no effect, reinforcing the independence from emotional processing. This finding is important, demostrating that emotional processing impairments in psychopaths might occur only when emotions are relevant to their task.

The elimination of the psychopathy effect on distance estimation when the Avatar was replaced with a non—anthropomorphic object is both novel and significant. Psychopathy is characterized by deficits in interpersonal behaviour, making social processing a core aspect of its manifestations^46,47^. The current work clearly demonstrates that the human-like appearance of an agent is essential for activating psychopathic traits, even in basic cognitive tasks such as distance estimation and underscores the importance of human resemblance in psychopathy. While focused on fundamental processes like distance estimation, the results highlight their social specificity, as the effects depended on the human-like features of the avatar. This suggests that even basic spatial cognition is influenced by social factors, linking spatial and social processing.

The simplified “social” nature of the avatar, featuring a mannequin with a pasted face and non-biological motion, may have influenced how participants perceived it as a social stimulus. This design, intended to prevent step-counting strategies, could have led participants to rely on visual cues, such as face visibility, rather than true social resemblance. Additionally, psychopathy-related traits may have affected whether the avatar was interpreted as social. Future work with more biologically dynamic stimuli or varied starting positions could help disentangle these effects.

Self-selection and practice effects (avatar task always before the cylinder task) are unlikely to explain the null results in the cylinder task. The long interval between avatar and cylinder experiments eliminated carry-over effects, and both experiments included extensive training to ensure familiarity. The PPI distribution was consistent across cohorts, and the psychopathy effect in the avatar task remained unchanged for the cylinder subset. These findings suggest self-selection did not bias the results.

After identifying the positive offset bias of the distance estimation error, attributed to the VR method used, it was apparent that in the Avatar case the effect of psychopathy switches from distance underestimation to overestimation along the PPI range. People at the lower part of psychopathy scale perceived that the Avatar had already reached the target distance when it was still further away (positive error, underestimation). People at the higher end of psychopathy scale perceived that the agent is at the target distance when it had already moved past it and was situated nearer to them (negative error, overestimation). People with average psychopathy levels perceived distance undistorted, around its actual value.

The observed distance estimation bias, characterized by consistent underestimation of distance for approaching stimuli (e.g. estimating 1.1 m as 1 m ), aligns with established findings in the literature on dynamic distance perception. Looming stimuli, such as approaching objects, are often perceived as closer than they truly are due to their behavioral urgency, an evolutionary adaptation for threat detection^48–52^. Conversely, receding stimuli are often perceived as farther away, reflecting complementary biases in distance estimation^53^. These findings suggest that the underestimation bias observed in our study may stem from the inherent perceptual properties of looming stimuli, rather than the VR method bias we assumed. However, additional experiments would be necessary to confirm this explanation.

The effect of psychopathy on distance estimation was shown to be driven solely by the scale Self-centered Impulsivity (PPI-SI). This is a component of the dual process model of psychopathy^29^, which quantifies tendencies towards impulsivity, disinhibition, poor behavioral control, and egocentricity. The current results cannot be explained by generic impulsive behavior or lack of inhibition as in such case, high PPI-SI participants would stop the avatar prematurely, contrary to what was observed. Additionally in such cases the effect would also be present with the non-human-resembling object. Lilienfeld and Widows^23^ described PPI-SI traits as a “self-centered propensity to take advantage of others and to act on one’s impulses whenever deemed convenient.” Under this light, it is more likely that the current results capture the egocentric tendency to assert control on others. The individuals who have a more self-centered predisposition, with a tendency to take advantage of others for their own interests, let a human-resembling agent come closer, while still having the perception that it is situated further. The individuals with the opposite predisposition, an aversion towards taking advantage of others, keep a human-resembling agent further while still perceiving it as being closer. People with average social predisposition towards others don’t seem to experience such spatial transformations. So the effect of psychopathy on distance estimation appears to be an involuntary transformation of spatial perception based on the social predisposition of an individual towards others. Previous work by Witt, Proffitt, and Epstein^54^ has emphasized the role of effort and intent in shaping spatial judgments, proposing that individuals perceive distances differently based on their goals and anticipated effort. The heightened self-centered orientation characteristic of PPI-SI may similarly bias distance estimation by prioritizing goal-driven interpretations of socially salient stimuli.

The parts of the brain that are likely implicated in such spatial transformation of estimated distance by psychopathic traits, include areas involved the perception of egocentric space, like Posterior parietal cortex^55^ and Premotor Cortex^56^, areas involved in the identification of Human resemblance like Fusiform Gyrus^57^ and Extrastriate Body Area^58^ and areas involving social interactions such as Temporoparietal Junction^59^, Medial Prefrontal Cortex^60^ and Posterior Superior Temporal Sulcus^61^. Some of these areas have already been found to be involved in the modulation of personal space by various factors^62,63^.

In conclusion, this study advances our understanding of how social predispositions influences the perception of basic spatial parameters and underscores the critical role of human resemblance in the elicitition of these effects. By identifying Self-Centered Impulsivity as the main driving factor, this work elucidates a specific mechanism through which psychopathy affects spatial perception, bridging the gap between social and cognitive processes. As the traits captured by Self-Centered Impulsivity are not exclusive to psychopathy, these findings potnentially extend to other disorders and phenomena involving social interactions. This recognition broadens the applicability of the results, potentially informing new ways of diagnosis and interventions. Finally, these insights provide new threads for studying and understanding social behaviour not only in real but also in virtual settings, particularly as interactions with virtual agents, whether human or non-human, become increasingly prevalent in modern life.

## Methods

### Overview

The study was performed in three different stages. In stage 1, a large cohort of participants were invited to fill in online the Psychopathic Personality Inventory-Revised [PPI-R] questionnaire. In stage 2 the participants were invited to perform a Distance Estimation experiment to an approaching Avatar in a virtual environment. The Avatar had either a happy, angry or neutral facial expression. In Stage 3 participants were reinvited to perform an experiment similar to stage 2 but with the Avatar replaced by an object, which did not resemble a human form.

### Avatar facial expression stimuli

The emotional faces used for the Avatar experiment were taken from the “Karolinska Directed Emotional Faces” database^64^. The actors used in this database were between 20 and 30 years old. For the current study, a set of 48 actors (24 male and 24 female) was selected, each with the three face expressions, namely happy, angry, and neutral. Figure 1C shows the images for the three different emotions for the same actress from the database.

### Animation and online deployment

The main theme of the experiment was the estimation of distance to an approaching Avatar or object. This dictated the need for a realistic representation of distance, therefore, the experiment was designed in a three-dimensional virtual environment, displayed on a monitor. Two widely used animation software programs, Blender^65^ and Unity^66^, were used for this task. The online deployment was performed through a JATOS server^67^.

#### Blender

Blender was used for the design of the animated Avatar and non-human-resembling object. The Avatar was designed in a simple box-shaped style (see Fig. 2D) and was rigged with a basic human skeleton template of Blender. The height of the avatar was 1.73 m and its head had a cuboid shape. The face images from the KDEFdatabase were attached as textures to the front facet of the head cuboid. In the case of non-human-resembling object, the exact object used was a Cylinder, shown in Fig. 2D next to the Avatar. The cylinder had the exact same height as the avatar and its diameter was equal to the width of its cuboid head.

The translational movement of the Avatar was also animated in Blender as a simple translation from its starting position, without any walking movements of the legs and arms in order to avoid the risk of the participants estimating distance by counting leg or foot movement cycles. Blender was also used to design a fixation mark, a panel for displaying the target distance to the participant and a panel for providing feedback to the participant, which can be seen in Fig. 2A, B.

#### Unity

The experiment was designed in Unity, one of the most popular game design software programs, based on the C# programming language. The animation components built in Blender were imported into Unity, where they were placed in the default 3D environment. A default virtual camera and light source were placed at a distance of 5.5 m away from the starting position of the Avatar and Cylinder. The animated translation was also imported into Unity and was directed towards the virtual camera. The camera view was used as the first-person view of the participants. A plane surface was added, covering the upper part of the screen, which was used to give instructions to the participants. After the entire experiment was designed and tested in Unity, it was compiled in WebGL format so it could be run online on the participant’s preferred web browser. All participants received an email with the link to a web-server (JATOS) hosting the experiment. The experiment was automatically deployed in full-screen mode to ensure maximum possible visibility of the stimulus.

#### JATOS

JATOS (“Just Another Tool for Online Studies”) is an open-source server-side (back-end) tool, specifically developed to help researchers set up and run online experiments on their own servers. The WebGL-version of the experiment was imported into the JATOS server, and then a common weblink was created which pointed to the experiment. Multiple participants could connect to the JATOS server and do the experiment in parallel. The JATOS API library is written in JavaScript and offers various functions, which can be used to upload data of the participant’s responses from the web browser running the experiment to the JATOS server from which it was launched. Unity, in which scripting is exclusively in C#, offers a way to call such JavaScript functions by creating some intermediate JavaScript functions inside a .jslib-file in the “Plugins” subfolder of the assets folder of the experiment.

### Psychopathy questionnaire

The Psychopathic Personality Inventory-Revised [PPI-R]^23^ focuses on eight characteristics of psychopathy measured with eight subscales. These subscales, with one exception, load on two higher order factors. Social influence, stress immunity, and fearlessness can be consolidated as “Fearless Dominance”. This factor represents emotional and interpersonal deficits and is also related to positive adjustment, such as charming behaviour(e.g., “Even when others are upset with me, I can usually win them over with my charm”). The characteristics machiavellian egocentricity, rebellious nonconformity, blame externalization, and carefree non-planfulness can be summarized as “Self-Centered Impulsivity”. This factor is related to disinhibition and impulsive behaviour(e.g., “I like to act first and think later”). Apart from these two main components the PPI-R also includes the characteristic Coldheartedness, which is associated with low empathy, lack of compassion and indifference towards other people’s feelings (e.g., “When someone is hurt by something I say or do, that’s their problem”)^68^.

To measure psychopathy, a short version of the Psychopathic Personality Inventory-Revised (PPI-R) was used^69^. This short version of the PPI-R is a self-assessment questionnaire that consists of 40 statements, which have to be rated on a 4-point rating scale with the categories wrong, rather wrong, rather correct or correct. The normalization of the questionnaire is based on a non-clinical student sample but can also be used for an older population^68^. The scores of all subscales were summed up to generate an overall psychopathy score, with higher scores indicating higher extent of psychopathy.

### Design and procedure of the main avatar experiment

The main experiment consisted of two parts. Participants first had to rate face expressions, and subsequently they had to complete a distance estimation task after a training session. The duration of the whole experiment was around 30 min. For participating in the experiment, the use of a computer or laptop instead of a tablet computer or smartphone was mandatory.

#### Rating face expressions

Participants were asked to rate a randomly selected set of 24 faces from the actual experiment (8 happy, 8 neutral and 8 angry face expressions). The set was different for each participant. The number of male and female actors in the rating set were equal. The rating scale consisted of 9 points, ranging from “negative” to “positive”. The middle rating point was labeled as “neutral” (see Fig. 1D).

#### Distance estimation

In the main task, participants were asked to estimate a target distance between an approaching Avatar and themselves. In the beginning of each trial, the target distance was shown in the middle of the screen as a decimal number in meters (Fig. 2A). The starting distance of the avatar from the participant was always 5.5 m. After a button press by the participant, the target distance display panel disappeared and the avatar appeared at the starting location with a fixation marker on its face. After 0.5 s the fixation marker disappeared and the avatar started “walking” towards the participant. The walking speed of the avatar was either slow (1.25 m/s) or fast (1.75 m/s). The participant’s task was to stop the avatar in the cued target distance as accurately as possible. In the instruction, participants were reminded to focus on the avatar’s face at any time.

The target distance in each trial was selected from six possible different locations with respect to the participant, ranging from 0.5 m to 3.6 m. In order to motivate the participants paying attention to the exact cued distance, two different sets of slightly different distances for each location were employed. Distance set A ([0.5, 1.1, 1.7, 2.3, 2.9, 3.5] meters) and distance set B ([0.6, 1.2, 1.8, 2.4, 3.0, 3.6] meters). One distance set was always used for trials of one speed and the other set for the other speed. The exact type of distance-set allocated to each speed was randomized across subjects. For half the subjects the allocation was distance set A – slow speed, and distance set B – fast speed, while for the other half vice versa.

The main part of the experiment was divided into four blocks, with each block consisting of 36 trials. This resulted in 144 trials in total. The number of trials per block for each of the three emotional face expressions was the same, namely 12. The facial identities used for these trials were randomly selected, without replacement from all possible 144 facial identity-emotion combinations. After each block, participants received feedback about their average error within this block as a decimal number (See Fig. 2B). If the avatar had been stopped on average within a range of +/− 0.5 m of the target distance, the feedback was labeled as “spot on”. If the avatar had been stopped closer or further than 0.5 m to the target distance, participants received the feedback “too close” or “too far”, respectively. A positive error value referred to distance underestimation and a negative error value referred to overestimation, as intuitively described in Fig. 2C.

#### Training

Right after the Face rating part and just before the main part of the Avatar experiment, participants underwent a training procedure. The main aim of this procedure was to make the participants familiar with the virtual environment and to train their perception of the different distances in it. The trial structure in this training was identical to the main part of the experiment, as described above. An avatar with a single neutral face expression, not used in the main experiment, was used in all the trials. The avatar walked always with the slow speed (1.25 m/s). The training session consisted of 40 trials and could be repeated by the participant if desired. Whereas in the main experiment, participants received feedback only after each block, in the training session feedback was given after each single trial (See Fig. 2B), in order to assist learning. There were five different target distances in the training, namely 0.5, 1, 2, 3 and 4 m. Each of these distances was used in 8 trials, in a shuffled sequence. The trials sequence was identical for all participants.

### Design and procedure of the cylinder experiment

The participants who performed the Avatar experiment were reinvited at a later time to perform a second follow-up experiment, in which the Avatar was replaced by an object, which did not resemble a human form. The aim of the experiment was twofold. First to investigate whether human resemblance is a crucial factor on the way that psychopathy affects the perception of interpersonal distance. And second to identify whether there is a bias in the distance estimation error due to the virtual environment used.

The exact object used was a cylinder, shown in Fig. 2D next to the Avatar. The cylinder had the exact same height as the avatar and its diameter was equal to the width of its head.

The design of this control experiment was based on the design of Avatar experiment with some important differences. As the moving stimulus was a cylinder without any facial expression, there was no parameterization of the factor “emotion”. The object moved with two different speeds, as in the Avatar experiment, and the target distances were also identical.

The two different speeds and six different distances led to 12 different unique trial cases. Each of this case was repeated 8 times, giving a total number of 96 trials. These were divided randomly in 4 blocks with 24 trials per block.

The structure of each trial was identical to the avatar experiment, as described earlier and depicted in Fig. 2A, B. The training session prior to the experiment was also identical to that of the Avatar. Finally identical was also the feedback about the distance error that was provided to the participants after each trial in the training session and after each block in the experiment sessions. The speed-distance-set allocation for a given participant was the same as in the Avatar case.

As emotion was not a factor in this control experiment, there was no Face Expression Rating session at the beginning of the experiment.

### Participants

#### Questionnaire

A total of 336 participants were recruited to complete the PPI-R questionnaires online. Participants were recruited from the participant pool of the Max Planck Isntitute for Empirical Aesthetics. They ranged in age from 18 to 77 years (M = 30.54, SD = 11.67). Of these, 251 identified as female, 82 as male, 1 selected “other,” and 3 declined to disclose their gender.

#### Avatar experiment

All participants were invited again for the experiment of distance estimation to an approaching Avatar. The experiment was performed in a Virtual Environment implemented on an online server. From the initial cohort, 128 participants accepted the invitation, 85 women, with an age range of 18 to 72 years (*M* = 30.61, *SD* = 12.41). Based on their scores in the PPI-R, participants were divided into three groups of low, middle and high psychopathic individuals. Using terciles (divided at 33.3% and 66.6%), participants with a psychopathy score of 81 or lower were labeled as low, with a psychopathy score of 92 or higher were labeled as high, and participants in between were labeled as middle psychopaths. The terciles made these three groups balanced, with 42 (low), 43 (middle) and 43 (high) psychopathic individuals respectively.

#### Cylinder experiment

The participants that took part in Avatar experiment were reinvited to participate in the Cylinder experiment. The time period between the two experiments was 21 months. In total 53 of them accepted the invitation. They were allocated to the low, middle and high psychopathy groups based on the same PPI-R threshold values as in the Avatar experiment. The allocation was 17 in the low, 19 in the middle and 17 in the high psychopathy group.

#### Estimation of sample size

The required sample size for the distance estimation experiment was computed using power analysis based on an effect size guided by previous closely-related work (see Supplementary Information). The analysis was based on a mixed-design ANOVA with an effect size of (f = 0.45), an alpha level of 0.05, a power level and three levels each for the between- and within-subject factors. The nonsphericity correction was set to 0.75. The analysis indicated that a total sample size of *N* = 36 participants would be required to detect an effect. A total of 110 participants agreed to take part in the distance estimation experiment with the avatar, far exceeding the minimum requirement of 36 participants established by the a priori power analysis. Of these, 52 participants also agreed to participate in the follow-up experiment, where the avatar was replaced by a cylinder. This number was likewise substantially greater than the required sample size from the power analysis.

#### Ethics statement

The experiments were approved by the Ethics Council of the Max Planck Society and was conducted in accordance with the Declaration of Helsinki. Written informed consent was given by all participants before the experiment.

### Analysis

#### Face rating

The facial expression ratings were analyzed using ANOVA with psychopathy level (low, middle, high) as a factor to explore its effect on emotion perception. Prior to the ANOVA, Shapiro-Wilk tests were performed for mean ratings of all three conditions to test whether assumption of normality was valid. Results were significant in all cases, indicating that the data was not normally distributed, *p* < 0.001. However, because of similar sample sizes, violation of the normal distribution assumption was accepted. There was homogeneity of the error variances, as assessed by Levene’s test (*p* > 0.05).

#### Avatar distance estimation error - outliers

Outliers were determined in three steps. First, the optional comments of all participants at the end of the experiment were reviewed and possible problems with the correct presentation of the stimuli were identified. Four participants reported complications during the distance estimation task and were therefore excluded from further analysis. Subsequently, we searched for participants who had estimation errors of more than 1 m in more than 50% of all trials. This applied to one participant who therefore was also excluded. Last, all trials with error values larger than 1 m were discarded. For 18 participants this resulted in some of the emotion-speed-distance combinations having no trials. For this reason, these 13 subjects were excluded. The remaining sample included 110 participants (18–70 years, M = 29.6, SD = 11.5) of which the majority was female (67.3%).

#### Avatar distance estimation error - ANOVA

A mixed-design ANOVA was performed in order to investigate the effect of psychopathy and avatar facial emotion on distance estimation error. Facial expression was the within-subject factor(neutral, happy, angry), while psychopathy level (low, mid, high) acted as between-subject factor. Avatar speed(slow, fast) was used as a second within-subject factor due to its direct modulation of the distance estimation error.

There was homogeneity of the error variances, as assessed by Levene’s test (*p* > 0.05). Prior to the ANOVA, Shapiro-Wilk tests were performed on the dependent variable to verify whether the assumption of normality was valid. Results were significant, indicating that the data is not normally distributed, *p* < 0.05. However, repeated measure ANOVA is relatively robust against violations of the normal distribution assumption^70–72^, therefore no data transformation was performed prior to the analysis.

When the ANOVA sphericity assumption was violated (Mauchly’s test, *p* < 0.05), the Greenhouse–Geisser correction was applied^73^.

The ANOVA was performed in MATLAB^74^ using the fitrm() and ranova() functions. Post-hoc tests with multiple comparison correction were performed with the multcompare() function.

#### Avatar distance estimation error – polynomial models

To analyze the relationship between distance error $\:\varDelta\:\theta\:$ and psychopathy index PPI, without discretizing into groups according to terciles, two different types of models were tested. The first type was a linear model of the form $\:\varDelta\:\theta\:=\beta\:\cdot\:PPI+\gamma\:$. The second type was a quadratic model of the form $\:\varDelta\:\theta\:=a\cdot\:{PPI}^{2}+\beta\:\cdot\:PPI+\gamma\:$. Comparison between the goodness of fit between the two types of models was performed based on their $\:{R}^{2}$ .

Linear models were fitted in MATLAB using the fitlm() function with a least-squares method using Bisquare weighting for minimizing the effect of outliers^75^. The quadratic model was fitted using the fitnlm() function, also using Bisquare weighting. The 95% confidence intervals of both models across the range of PPI values was computed using the MATLAB predict() function.

#### Cylinder distance estimation error – outliers

The same data quality criteria as in the Avatar experiment were applied and this resulted in the dataset of one participant being excluded from further analysis.

#### Cylinder distance estimation error – ANOVA

A mixed-design ANOVA was performed in order to investigate the effect of psychopathy on distance estimation error. Psychopathy level (low, mid, high) was the between-subject factor and speed(slow, fast) the within-subject factor,

Similarly to the Avatar experiment, τhere was homogeneity of the error variances, as assessed by Levene’s test (*p* > 0.05) and Shapiro-Wilk indicated that the data was not normally distributed, *p* < 0.05. As for the Avatar data, repeated measure ANOVA was considered relatively robust against this violation of the normal distribution assumption, therefore no data transformation was performed prior to further analysis. When the ANOVA sphericity assumption was violated (Mauchly’s test, *p* < 0.05), the Greenhouse–Geisser correction was applied^73^.

#### Cylinder distance estimation error – polynomial models

Similarly to the avatar case the relationship between distance error $\:\varDelta\:\theta\:$ and psychopathy index PPI was further investigated by fitting two types of models. A linear and a quadratic one. The models were fitted with the exact same procedures and functions as in the Avatar case.

## Electronic supplementary material

Below is the link to the electronic supplementary material.

Supplementary Material 1

Supplementary Material 2

## Data Availability

The datasets generated and/or analyzed during the current study are available from the corresponding author upon reasonable request.
