# Supplementary material for: Social processing distorts physical distance perception
Source: Sci Rep. 2025 Feb 15;15:5669. doi: 10.1038/s41598-025-89935-9 (PMC11830008; doi:10.1038/s41598-025-89935-9)
Supplement: Supplementary file 2 — Supplementary Material 2 [file 41598_2025_89935_MOESM2_ESM.docx]

# Supplementary Information – “S”

## STATISTICAL POWER ANALYSIS

The effect size was calculated based on closely related work on preferred distance.

1. **Vieira and Abigail, 2013**

In this paper, the preferred distance of participants with high Coldheartedness index from an experimenter was 0.98 m while for low Coldheartedness was 0.67 m. So the difference in the preferred distance between the two groups was 0.31cm. The mean preferred distance across all 46 participants, for all groups of Coldheartedness, was 0.8m with Standard Deviation 0.3m.

So in this work the Effect Size was 0.98-0.67/0.3 = 1.033, a very large value.

1. **Welsch et al, 2018**

In this work 40 participants approached an avatar and stopped at a comfortable distance. The avatar had either a happy or an angry face. The average distance across all participants and all facial expressions was 1.18 m and the standard deviation 0.22m. The distance difference between the happy and angry cases about 10cm for all psychopathy levels. For low psychopathy group, where the difference was more pronounced, it was about 15.5 cm. So the Effect size in this work was 0.1/0.22 = 0.455 for all psychopathy levels and 0.7 for low psychopathy were the main effect was identified.

Guided by the above published work we decided to go for and effect size of 0.45, corresponding to a target minimum difference between conditions of 10 cm, with an overall standard deviation of distance estimation error of 22 cm. These values based on previous research covered our expectations regarding the differences in distance between facial expressions and between different psychopathy groups.

We performed a priori power analysis using G*Power. As we were interested in the effect of Psychopathy Group (low, middle, high) and Facial Emotion (happy, angry, neutral), the number of subjects required was estimated by the following settings:

Effect size: 0.45

We conducted an a priori power analysis using G*Power 3.1 (Faul et al., 2009) to determine the required sample size for a mixed-design ANOVA with one between-subject factor (psychopathy group: Low, Middle, High) and one within-subject factor (facial expression: Neutral, Angry, Happy). We specified an effect size of 0.45, an alpha level of 0.05 and a power level of 0.80. The correlation among repeated measures was set to r=0.5, and the nonsphericity correction was set to 0.75 to account for violations of sphericity. The analysis indicated that a total sample size of **N=36 participants** would be required to detect an effect of this magnitude. This number of participants was comparable to both the above mentioned studies used as guides for estimation of effect size.

As already described in the methods, prior to the distance estimation experiment, first an online questionnaire study was conducted with the largest pool of 366 participants, in order to get an accurate estimate of the distribution of psychopathy index, which was used to define the limits of the 3 psychopathy groups (low, medium, high). As the distance estimation experiment was also performed online, it was straightforward to reinvite all participants from the initial questionnaire phase mentioned above so that we can have as high statistical power as possible. The 110 participants that accepted to participate the distance estimation experiment with the avatar, where many more than the 36 minimum required based on our a priori power analysis. Out of these participants, 52 accepted to also participate in the follow-up experiment where the avatar was replaced by the cylinder. This number of participants was also significantly larger than the necessary number from the power analysis.

The adequacy of the 52 number of participants for capturing the effect of psychopathy on distance estimation error is also demonstrated in our results section regarding the Cylinder experiment. There the parametric models also for the Avatar case are fitted again, but this time only with the data from the 52 participants that also conducted the Cylinder experiment. The effect of psychopathy is still captured even with this lower number of participants and this was expected as it is well above the minimum number of required participants.

## ANOVA ANALYSIS TABLES - AVATAR and CYLINDER experiments

**AVATAR – WITHIN FACTORS: Emotion, Speed BETWEEN FACTORS: PPI-Group**

Table S1.1 – AVATAR experiment - ANOVA table for within-factors: Emotion, Speed and Between-factors: PPI-Group

| Effect | SumSq | DF | MeanSq | F | p-Value |
| --- | --- | --- | --- | --- | --- |
| (Intercept) | 58.109 | 1 | 58.109 | 63.909 | 1.63e-12 |
| PPIGROUP | 6.158 | 2 | 3.079 | 3.386 | 0.0375 |
| Error | 97.289 | 107 | 0.909 |  |  |
| (Intercept):EMOs | 0.00427 | 2 | 0.00214 | 0.0679 | 0.9344 |
| PPIGROUP:EMOs | 0.1363 | 4 | 0.03407 | 1.083 | 0.3657 |
| Error(EMOs) | 6.730 | 214 | 0.03145 |  |  |
| (Intercept):SPDs | 11.479 | 1 | 11.479 | 159.69 | 5.97e-23 |
| PPIGROUP:SPDs | 0.09345 | 2 | 0.04672 | 0.650 | 0.5241 |
| Error(SPDs) | 7.692 | 107 | 0.07188 |  |  |
| (Intercept):EMOs:SPDs | 0.0514 | 2 | 0.02568 | 0.869 | 0.4210 |
| PPIGROUP:EMOs:SPDs | 0.1414 | 4 | 0.03535 | 1.196 | 0.3137 |
| Error(EMOs:SPDs) | 6.328 | 214 | 0.02957 |  |  |

**CYLINDER – WITHIN FACTORS: Speed BETWEEN FACTORS: PPI-Group**

Table S1.2 – CYLINDER experiment - ANOVA table for within-factors: Speed and Between-factors: PPI-Group

| Effect | SumSq | DF | MeanSq | F | p-Value |
| --- | --- | --- | --- | --- | --- |
| (Intercept) | 6.313 | 1 | 6.313 | 19.996 | 4.61e-05 |
| PPIGROUP | 0.308 | 2 | 0.154 | 0.488 | 0.6167 |
| Error | 15.470 | 49 | 0.316 |  |  |
| (Intercept):SPDs | 2.325 | 1 | 2.325 | 64.508 | 1.71e-10 |
| PPIGROUP:SPDs | 0.113 | 2 | 0.056 | 1.564 | 0.2196 |
| Error(SPDs) | 1.766 | 49 | 0.036 |  |  |

**AVATAR - POST-HOC TESTS on factor EMOTION (Bonferonni correction)**

Table S1.3 – AVATAR experiment – POST-HOC tests between pairs of Emotion

| Emotion 1 | Emotion2 | Difference | StdErr | pValue | Lower | Upper |
| --- | --- | --- | --- | --- | --- | --- |
| Neutral | **Angry** | -0.0026 | 0.0066 | 1.0000 | -0.0187 | 0.0136 |
| Neutral | **Happy** | -0.0010 | 0.0075 | 1.0000 | -0.0192 | 0.0172 |
| Angry | **Neutral** | 0.0026 | 0.0066 | 1.0000 | -0.0136 | 0.0187 |
| Angry | **Happy** | 0.0016 | 0.0068 | 1.0000 | -0.0151 | 0.0182 |
| Happy | **Neutral** | 0.0010 | 0.0075 | 1.0000 | -0.0172 | 0.0192 |
| Happy | **Angry** | -0.0016 | 0.0068 | 1.0000 | -0.0182 | 0.0151 |

## EFFECT OF SPEED on DISTANCE ESTIMATION ERROR

**LINEAR MODELS of DISTANCE ERROR vs PPI for different Avatar SPEEDS**


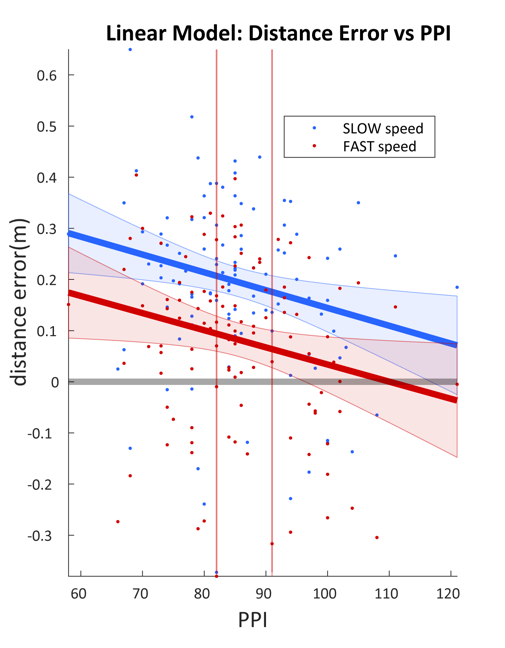


Figure S1. Linear Model of Distance error vs PPI for different Avatar speeds. Linear fit between PPI and Mean Distance Error of each participant. The shaded area around the fitted line represents the 95% confidence area of the fit. With blue color are depicted the data points, linear model and confidence area for the trials when the Avatar was moving with SLOW speed. And with red color when the Avatar was moving with FAST speed. The main difference between the two speeds is the offset, while the slopes remain identical.

## EFFECT OF DISTANCE on DISTANCE ESTIMATION ERROR

### ANOVA TABLES

**AVATAR – WITHIN FACTORS: Emotion, Speed and DISTANCE, BETWEEN FACTORS: PPI-Group**

Table S2.1- AVATAR experiment - ANOVA table for within-factors: Emotion, Speed, Distance and Between-factors: PPI-Group

| Effect | SumSq | DF | MeanSq | F | p-Value |
| --- | --- | --- | --- | --- | --- |
| (Intercept) | 58.109 | 1 | 58.109 | 63.909 | 1.63e-12 |
| PPIGROUP | 6.158 | 2 | 3.079 | 3.386 | 0.0375 |
| Error | 97.289 | 107 | 0.909 |  |  |
| (Intercept):EMOs | 0.00427 | 2 | 0.00214 | 0.0679 | 0.9344 |
| PPIGROUP:EMOs | 0.1363 | 4 | 0.03407 | 1.083 | 0.3657 |
| Error(EMOs) | 6.730 | 214 | 0.03145 |  |  |
| (Intercept):SPDs | 11.479 | 1 | 11.479 | 159.69 | 5.97e-23 |
| PPIGROUP:SPDs | 0.09345 | 2 | 0.04672 | 0.650 | 0.5241 |
| Error(SPDs) | 7.692 | 107 | 0.07188 |  |  |
| (Intercept):DISTs | 22.288 | 5 | 4.4575 | 24.186 | 5.68e-22 |
| PPIGROUP:DISTs | 1.596 | 10 | 0.1596 | 0.866 | 0.5652 |
| Error(DISTs) | 98.600 | 535 | 0.1843 |  |  |
| (Intercept):EMOs:SPDs | 0.0514 | 2 | 0.02568 | 0.868 | 0.4210 |
| PPIGROUP:EMOs:SPDs | 0.1414 | 4 | 0.03535 | 1.196 | 0.3137 |
| Error(EMOs:SPDs) | 6.328 | 214 | 0.02957 |  |  |
| (Intercept):EMOs:DISTs | 0.2902 | 10 | 0.02902 | 0.899 | 0.5331 |
| PPIGROUP:EMOs:DISTs | 0.5425 | 20 | 0.02712 | 0.841 | 0.6644 |
| Error(EMOs:DISTs) | 34.521 | 1070 | 0.03226 |  |  |
| (Intercept):SPDs:DISTs | 0.8844 | 5 | 0.17688 | 4.916 | 0.0002 |
| PPIGROUP:SPDs:DISTs | 0.2147 | 10 | 0.02147 | 0.597 | 0.8170 |
| Error(SPDs:DISTs) | 19.250 | 535 | 0.03598 |  |  |
| (Intercept):EMOs:SPDs:DISTs | 0.0784 | 10 | 0.00784 | 0.252 | 0.9904 |
| PPIGROUP:EMOs:SPDs:DISTs | 0.4056 | 20 | 0.02028 | 0.653 | 0.8736 |
| Error(EMOs:SPDs:DISTs) | 33.237 | 1070 | 0.03106 |  |  |

**CYLINDER – WITHIN FACTORS: Emotion, Speed and DISTANCE, BETWEEN FACTORS: PPI-Group**

Table S2.2- CYLINDER experiment - ANOVA table for within-factors: Speed, Distance and Between-factors: PPI-Group

| Effect | SumSq | DF | MeanSq | F | p-Value |
| --- | --- | --- | --- | --- | --- |
| (Intercept) | 6.313 | 1 | 6.313 | 19.996 | 4.61e-05 |
| PPIGROUP | 0.308 | 2 | 0.154 | 0.488 | 0.6167 |
| Error | 15.470 | 49 | 0.316 |  |  |
| (Intercept):SPDs | 2.325 | 1 | 2.325 | 64.508 | 1.71e-10 |
| PPIGROUP:SPDs | 0.1127 | 2 | 0.05637 | 1.564 | 0.2196 |
| Error(SPDs) | 1.766 | 49 | 0.03605 |  |  |
| (Intercept):DISTs | 1.2166 | 5 | 0.24331 | 2.755 | 0.0193 |
| PPIGROUP:DISTs | 1.560 | 10 | 0.156 | 1.766 | 0.0674 |
| Error(DISTs) | 21.639 | 245 | 0.08832 |  |  |
| (Intercept):SPDs:DISTs | 0.1820 | 5 | 0.03641 | 2.291 | 0.0464 |
| PPIGROUP:SPDs:DISTs | 0.2041 | 10 | 0.02041 | 1.284 | 0.2396 |
| Error(SPDs:DISTs) | 3.894 | 245 | 0.01589 |  |  |

**AVATAR - POST-HOC TESTS on factor EMOTION PER DISTANCE (Bonferonni correction)**

Table S2.3 – AVATAR experiment – POST-HOC tests between pairs of Emotion at EACH DISTANCE LOCATION

| DISTs | EMOTION 1 | EMOTION 2 | Difference | StdErr | p-value | Lower CI | Upper CI |
| --- | --- | --- | --- | --- | --- | --- | --- |
| D1 | NEUTRAL | ANGRY | 0.0092 | 0.0099 | 1.000 | -0.0150 | 0.0333 |
| D1 | NEUTRAL | HAPPY | 0.0049 | 0.0105 | 1.000 | -0.0207 | 0.0304 |
| D1 | ANGRY | HAPPY | -0.0043 | 0.0112 | 1.000 | -0.0316 | 0.0230 |
| D2 | NEUTRAL | ANGRY | 0.0165 | 0.0153 | 0.843 | -0.0206 | 0.0536 |
| D2 | NEUTRAL | HAPPY | 0.0135 | 0.0136 | 0.964 | -0.0195 | 0.0465 |
| D2 | ANGRY | HAPPY | -0.0030 | 0.0157 | 1.000 | -0.0411 | 0.0351 |
| D3 | NEUTRAL | ANGRY | -0.0218 | 0.0164 | 0.559 | -0.0615 | 0.0180 |
| D3 | NEUTRAL | HAPPY | 0.0119 | 0.0188 | 1.000 | -0.0339 | 0.0577 |
| D3 | ANGRY | HAPPY | 0.0337 | 0.0180 | 0.193 | -0.0101 | 0.0774 |
| D4 | NEUTRAL | ANGRY | -0.0008 | 0.0223 | 1.000 | -0.0551 | 0.0535 |
| D4 | NEUTRAL | HAPPY | -0.0261 | 0.0209 | 0.645 | -0.0769 | 0.0248 |
| D4 | ANGRY | HAPPY | -0.0252 | 0.0170 | 0.422 | -0.0666 | 0.0161 |
| D5 | NEUTRAL | ANGRY | -0.0012 | 0.0211 | 1.000 | -0.0524 | 0.0501 |
| D5 | NEUTRAL | HAPPY | -0.0000 | 0.0201 | 1.000 | -0.0488 | 0.0488 |
| D5 | ANGRY | HAPPY | 0.0012 | 0.0203 | 1.000 | -0.0483 | 0.0506 |
| D6 | NEUTRAL | ANGRY | -0.0173 | 0.0185 | 1.000 | -0.0622 | 0.0277 |
| D6 | NEUTRAL | HAPPY | -0.0102 | 0.0169 | 1.000 | -0.0512 | 0.0308 |
| D6 | ANGRY | HAPPY | 0.0071 | 0.0182 | 1.000 | -0.0371 | 0.0512 |

**POST HOC TESTS between Distances for AVATAR for each SPEED**

### AVATAR - SLOW Speed

Table S2.4 – AVATAR experiment – POST-HOC tests between pairs of Distances for Slow Speed

| **DISTs_1** | **DISTs_2** | **Difference** | **StdErr** | **pValue** | **Lower** | **Upper** |
| --- | --- | --- | --- | --- | --- | --- |
| D1 | **D2** | -0.1413 | 0.0192 | 6.24×10−10 | -0.1990 | -0.0836 |
| D1 | D3 | -0.0331 | 0.0254 | 1.0000 | -0.1092 | 0.0431 |
| D1 | D4 | -0.0079 | 0.0258 | 1.0000 | -0.0853 | 0.0695 |
| D1 | D5 | -0.0478 | 0.0317 | 1.0000 | -0.1429 | 0.0474 |
| D1 | D6 | 0.0784 | 0.0348 | 0.3939 | -0.0260 | 0.1828 |
| D2 | **D3** | 0.1082 | 0.0180 | 4.10×10−7 | 0.0541 | 0.1624 |
| D2 | **D4** | 0.1334 | 0.0216 | 1.83×10−7 | 0.0685 | 0.1983 |
| D2 | **D5** | 0.0935 | 0.0296 | 0.0303 | 0.0048 | 0.1823 |
| D2 | **D6** | 0.2197 | 0.0337 | 3.76×10−8 | 0.1183 | 0.3210 |
| D3 | D4 | 0.0252 | 0.0225 | 1.0000 | -0.0423 | 0.0926 |
| D3 | D5 | -0.0147 | 0.0290 | 1.0000 | -0.1018 | 0.0724 |
| D3 | **D6** | 0.1114 | 0.0330 | 0.0154 | 0.0123 | 0.2106 |
| D4 | D5 | -0.0399 | 0.0230 | 1.0000 | -0.1089 | 0.0292 |
| D4 | **D6** | 0.0863 | 0.0267 | 0.0245 | 0.0061 | 0.1664 |
| D5 | **D6** | 0.1261 | 0.0187 | 1.31×10−8 | 0.0699 | 0.1824 |

### AVATAR - FAST Speed

Table S2.5 – AVATAR experiment – POST-HOC tests between pairs of Distances for Fast Speed

| DISTs_1 | DISTs_2 | Difference | StdErr | pValue | Lower | Upper |
| --- | --- | --- | --- | --- | --- | --- |
| D1 | **D2** | -0.1392 | 0.0195 | 1.94×10−9 | -0.1979 | -0.0805 |
| D1 | D3 | 0.0057 | 0.0236 | 1.0000 | -0.0653 | 0.0767 |
| D1 | D4 | 0.0392 | 0.0266 | 1.0000 | -0.0407 | 0.1192 |
| D1 | D5 | 0.0152 | 0.0314 | 1.0000 | -0.0790 | 0.1095 |
| D1 | **D6** | 0.1617 | 0.0325 | 3.80×10−5 | 0.0641 | 0.2593 |
| D2 | **D3** | 0.1449 | 0.0159 | 7.68×10−14 | 0.0972 | 0.1926 |
| D2 | **D4** | 0.1784 | 0.0215 | 5.28×10−12 | 0.1138 | 0.2430 |
| D2 | **D5** | 0.1544 | 0.0294 | 1.14×10−5 | 0.0662 | 0.2427 |
| D2 | **D6** | 0.3009 | 0.0322 | 2.24×10−14 | 0.2043 | 0.3975 |
| D3 | D4 | 0.0335 | 0.0203 | 1.0000 | -0.0274 | 0.0944 |
| D3 | D5 | 0.0095 | 0.0266 | 1.0000 | -0.0705 | 0.0895 |
| D3 | **D6** | 0.1560 | 0.0296 | 1.06×10−5 | 0.0671 | 0.2448 |
| D4 | D5 | -0.0240 | 0.0210 | 1.0000 | -0.0869 | 0.0389 |
| D4 | **D6** | 0.1225 | 0.0264 | 1.49×10−4 | 0.0432 | 0.2018 |
| D5 | **D6** | 0.1465 | 0.0184 | 3.00×10−11 | 0.0912 | 0.2017 |

**POST HOC TESTS between Distances for CYLINDER for each SPEED**

### CYLINDER SLOW Speed

Table S2.6 – CYLINDER experiment – POST-HOC tests between pairs of Distances for Slow Speed

| **DISTs_1** | **DISTs_2** | **Difference** | **StdErr** | **pValue** | **Lower** | **Upper** |
| --- | --- | --- | --- | --- | --- | --- |
| D1 | D2 | -0.0794 | 0.0316 | 0.231 | -0.1769 | 0.0182 |
| D1 | D3 | 0.0531 | 0.0404 | 1.000 | -0.0716 | 0.1779 |
| D1 | D4 | -0.0416 | 0.0440 | 1.000 | -0.1773 | 0.0940 |
| D1 | D5 | -0.1247 | 0.0489 | 0.209 | -0.2755 | 0.0262 |
| D1 | D6 | -0.0044 | 0.0504 | 1.000 | -0.1598 | 0.1511 |
| D2 | **D3** | 0.1325 | 0.0353 | 0.007 | 0.0235 | 0.2415 |
| D2 | D4 | 0.0377 | 0.0428 | 1.000 | -0.0944 | 0.1698 |
| D2 | D5 | -0.0453 | 0.0520 | 1.000 | -0.2058 | 0.1152 |
| D2 | D6 | 0.0750 | 0.0543 | 1.000 | -0.0927 | 0.2426 |
| D3 | D4 | -0.0948 | 0.0451 | 0.611 | -0.2339 | 0.0444 |
| D3 | **D5** | -0.1778 | 0.0538 | 0.027 | -0.3437 | -0.0119 |
| D3 | D6 | -0.0575 | 0.0557 | 1.000 | -0.2293 | 0.1143 |
| D4 | D5 | -0.0830 | 0.0391 | 0.585 | -0.2038 | 0.0378 |
| D4 | D6 | 0.0373 | 0.0409 | 1.000 | -0.0889 | 0.1634 |
| D5 | **D6** | 0.1203 | 0.0270 | 0.001 | 0.0371 | 0.2035 |

### CYLINDER - FAST Speed

Table S2.7 – CYLINDER experiment – POST-HOC tests between pairs of Distances for Fast Speed

| **DISTs_1** | **DISTs_2** | **Difference** | **StdErr** | **pValue** | **Lower** | **Upper** |
| --- | --- | --- | --- | --- | --- | --- |
| D1 | D2 | -0.0564 | 0.0276 | 0.699 | -0.1417 | 0.0289 |
| D1 | D3 | 0.0532 | 0.0380 | 1.000 | -0.0642 | 0.1706 |
| D1 | D4 | 0.0090 | 0.0432 | 1.000 | -0.1241 | 0.1422 |
| D1 | D5 | -0.0237 | 0.0495 | 1.000 | -0.1764 | 0.1290 |
| D1 | D6 | 0.0328 | 0.0552 | 1.000 | -0.1377 | 0.2032 |
| D2 | **D3** | 0.1096 | 0.0318 | 0.017 | 0.0115 | 0.2076 |
| D2 | D4 | 0.0654 | 0.0478 | 1.000 | -0.0822 | 0.2130 |
| D2 | D5 | 0.0327 | 0.0562 | 1.000 | -0.1406 | 0.2060 |
| D2 | D6 | 0.0892 | 0.0630 | 1.000 | -0.1051 | 0.2834 |
| D3 | D4 | -0.0441 | 0.0396 | 1.000 | -0.1662 | 0.0779 |
| D3 | D5 | -0.0769 | 0.0499 | 1.000 | -0.2310 | 0.0773 |
| D3 | D6 | -0.0204 | 0.0593 | 1.000 | -0.2035 | 0.1626 |
| D4 | D5 | -0.0327 | 0.0331 | 1.000 | -0.1350 | 0.0696 |
| D4 | D6 | 0.0237 | 0.0431 | 1.000 | -0.1093 | 0.1568 |
| D5 | D6 | 0.0564 | 0.0330 | 1.000 | -0.0454 | 0.1583 |

**MEAN DISTANCE ESTIMATION ERROR per DISTANCE LOCATION**

**
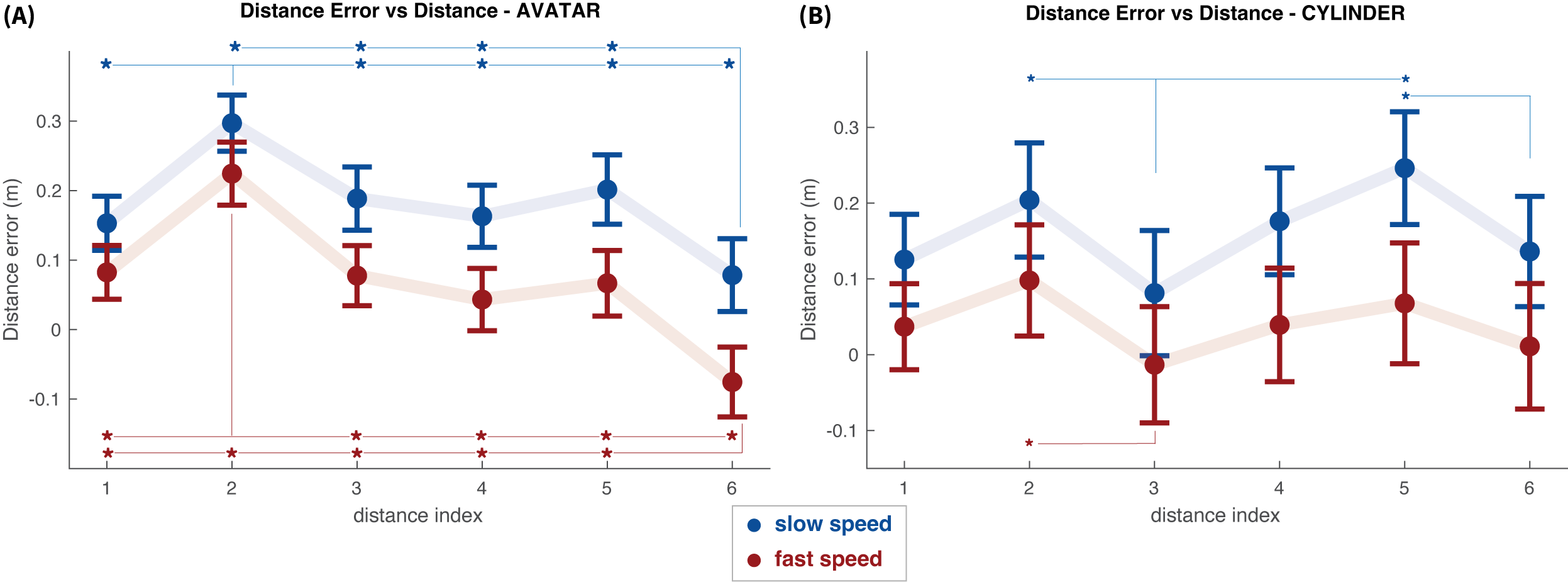
**

Figure S2. Distance Estimation Error at different distances. (A) Distance error vs distance for the AVATAR case. Six distance positions ([0.5 or 0.6, 1.1 or 1.2, 1.7 or 1.8, 2.3 or 2.4, 2.9 or 3.0, 3.5 or 3.6] meters). Results are presented separately for the two different speeds, slow(blue) and fast(red). The distance error is systematically higher across all distances for the slow speed. Distance error was comparable, and statistically non-significantly different, between 4 distance locations. The other 2 distance locations had significantly deviating errors. Location 2 (1.1 or 1.2 meters) had the highest estimation error than the others and this difference was significantly different with all other locations. Location 2 (1.1 or 1.2 meters) had the highest estimation error than the others and this difference was significantly different with all other locations. This is indicated by the significance markers over all locations, joined by a line starting from Location 2. Location 6 (3.5 or 3.6 meters) had the smallest estimation error than the others and this difference was significantly different with all other locations, as also indicated by the significance markers. The only exception is the difference with Location 1, with slow speed. (B) Distance error vs distance for the CYLINDER case. The distance error is systematically higher across all distances for the slow speed, as in the Avatar case. Between different locations, the distance estimation error was comparable in the vast majority of cases, with only few exceptions, marked with significance markers. This is contrast to the Avatar case where 2 locations had significantly different distance errors with almost all other locations.

## NOTE on the DIFFERENT NUMBER of TRIALS in the AVATAR and CYLINDER experiments

Both experiments were designed with identical structures, comprising four blocks, with feedback provided at the end of each block. In the avatar experiment, the within-subject factors included Facial Emotion (3 levels), Speed (2 levels), and Location (6 levels), resulting in 36 trials per block (3 trials per Speed-Location combination per block, or 12 trials across all blocks). In the cylinder experiment, only Speed (2 levels) and Location (6 levels) were included, resulting in 12 Speed-Location combinations per block. To ensure sufficient statistical power, each Speed-Location combination was repeated twice per block, totaling eight repetitions across the entire experiment.

From an analysis of variance (ANOVA) perspective, the cylinder experiment provided sufficient statistical power despite fewer trials. The cylinder condition had eight trials per Speed-Location combination, compared to the avatar condition's 12 trials, which were distributed across three emotions (four trials per Speed-Location-Emotion combination). In linear regression models, the avatar condition included 144 total trials per participant, while the cylinder condition included 96. This difference was unlikely to affect the estimation of the mean distance error, as the trials were evenly distributed across all Speed-Location conditions, ensuring the sample mean remained an unbiased estimator. The primary difference lay in the standard error of the mean, which was expected to be 22% higher in the cylinder condition ($\sqrt{144}/\sqrt{96}$ ≈1.22). This is reflected in the slightly wider confidence intervals for the cylinder model. However, these broader intervals did not impact the observed abolition of the psychopathy effect in the cylinder condition.
